# KSHV-Mediated Regulation of Par3 and SNAIL Contributes to B-Cell Proliferation

**DOI:** 10.1371/journal.ppat.1005801

**Published:** 2016-07-27

**Authors:** Hem C. Jha, Zhiguo Sun, Santosh K. Upadhyay, Darine W. El-Naccache, Rajnish K. Singh, Sushil K. Sahu, Erle S. Robertson

**Affiliations:** Department of Otorhinolaryngology-Head and Neck Cancer, and Tumor Virology Program and Tumor Virology Program, Abramson Cancer Center, Perelman School of Medicine at the University of Pennsylvania, Philadelphia, Pennsylvania, United States of America; University of North Carolina at Chapel Hill, UNITED STATES

## Abstract

Studies have suggested that Epithelial–Mesenchymal Transition (EMT) and transformation is an important step in progression to cancer. Par3 (partitioning-defective protein) is a crucial factor in regulating epithelial cell polarity. However, the mechanism by which the latency associated nuclear antigen (LANA) encoded by Kaposi's Sarcoma associated herpesvirus (KSHV) regulates Par3 and EMTs markers (Epithelial-Mesenchymal Transition) during viral-mediated B-cell oncogenesis has not been fully explored. Moreover, several studies have demonstrated a crucial role for EMT markers during B-cell malignancies. In this study, we demonstrate that Par3 is significantly up-regulated in KSHV-infected primary B-cells. Further, Par3 interacted with LANA in KSHV positive and LANA expressing cells which led to translocation of Par3 from the cell periphery to a predominantly nuclear signal. Par3 knockdown led to reduced cell proliferation and increased apoptotic induction. Levels of SNAIL was elevated, and E-cadherin was reduced in the presence of LANA or Par3. Interestingly, KSHV infection in primary B-cells led to enhancement of SNAIL and down-regulation of E-cadherin in a temporal manner. Importantly, knockdown of SNAIL, a major EMT regulator, in KSHV cells resulted in reduced expression of LANA, Par3, and enhanced E-cadherin. Also, SNAIL bound to the promoter region of p21 and can regulate its activity. Further a SNAIL inhibitor diminished NF-kB signaling through upregulation of Caspase3 in KSHV positive cells *in vitro*. This was also supported by upregulation of SNAIL and Par3 in BC-3 transplanted NOD-SCID mice which has potential as a therapeutic target for KSHV-associated B-cell lymphomas.

## Author Summary

Par3 (partitioning-defective protein) is characterized as a cell polarity protein with a crucial role in regulating EMT progression in KSHV-infected cells. Notably, SNAIL, a major EMT regulator is targeted through expression of Par3 and Latency associated nuclear antigen (LANA) during KSHV infection. In this study we show that LANA positively regulated Par3 and SNAIL while E-cadherin was inversely regulated during KSHV infection of B-cells. Furthermore, SNAIL binding with the potent kinase inhibitor p21 in KSHV positive cells enhanced NF-kB signaling. These results demonstrate the importance of Par3 and SNAIL in development of KSHV-induced B-cells cancers through regulation of EMT related antigens, and may be critical for driving KSHV-associated primary effusion lymphoma.

## Introduction

In 1994, Kaposi's sarcoma-associated herpesvirus (KSHV) was first identified [1]. Studies have shown that KSHV is predominantly involved in two kinds of cancers, originated through B-cells and endothelial cells [2,3]. In B-cells, it contributes to primary effusion lymphoma (PEL) as well as multicentric Castleman disease a rare form of lymphoproliferative disorder [4,5]. Kaposi sarcoma (KS) is derived from the lymphatic endothelial cell (LEC) lineage [6], and usually presents as colored reddish brown lesions or patches on the skin, and may spread to internal organs [7]. Additionally, other disease phenotypes like distal metastasis or pulmonary KS can be observed in AIDS-KS patients and causes symptoms like diffused lung disease [8]. B-cell neoplasias are also known to migrate into body cavities or internal organs [9]. Therefore KSHV infection is likely to be important for inducing cell migration, invasion and disease progression [10]. However, the mechanism by which cell migration, and cell invasion markers contribute to KSHV-mediated B-cell infection is still mostly unexplored.

It is known that KSHV lytic infection of endothelial cells leads to down-regulation of VE-cadherin protein levels [11]. Interestingly, KSHV induced degradation of VE-cadherin correlated with internalized virus particles [11]. Also, KSHV infection modulates the production of multiple MMPs to amplify cell invasiveness and thus adds to pathogenesis of KSHV-induced malignancies [12]. A fundamental process for unicellular and multicellular organisms is polarization, which is required for proper differentiation, proliferation and morphogenesis [13]. Formation of mesenchymal cells from epithelia was defined as Epithelial-Mesenchymal Transition (EMT). A hallmark of EMT is the loss or attenuation of epithelial polarity, which mainly occurs during metastasis and cancer progression [14,15]. In this process junction proteins localize differently in proliferating (mesenchyma) or (TJ) tight junction -containing epithelial cells, which may be suggestive of their specific functions [16]. EMT allows polarized epithelial cells to interact with the basement membrane through its basal surface, leading to biochemical changes that facilitate the mesenchymal cell phenotype. This enhances the migratory capacity, invasiveness, elevated resistance to apoptosis, and increased production of the ECM components [17,18]. Additionally, EMT is illustrated by disruption of epithelial junctions, altering of actin cytoskeleton, loss of cell polarity, variation of cell–matrix adhesion, and amplified cell motility [19]. EMT progresses in a step wise fashion, which starts with disruption of cell junctions and suppression of E-cadherin expression [20]. Recently, Lemma et al., showed the EMT like structure in B-cell lymphomas [21]. Also, Tilló et al., demonstrated that the EMT activator promotes tumor growth in mantle cell lymphoma [22]. Hence to study EMT markers in KSHV-induced B-cell lymphoma is crucial to explore.

Partitioning-defective (PARs) proteins represent a component of the body defense system, and aggressively participates in the inflammatory response [23]. PAR proteins were discovered in *C*. *elegans* [23]. More specifically, Par3 plays a crucial role in establishment and progression of epithelial cell polarity [24]. However, only specific stimuli are able to initiate the differentiation of epithelial cells to mesenchymal through genetic re-programming to form mesenchymal-like cells [25]. In another study, using cultured epithelial cells the Par3 complex supports the creation of epithelial cells tight junctions thereby adding significantly to the establishment and maintenance of apical–basal polarity [26].

In many cancer cell lines, SNAIL-1 and SNAIL-2 (Slug) are considered strong repressors of E-cadherin expression [27]. SNAIL-1 expression is enhanced in bladder cancer [28]. However, there were no significant relationship of SNAIL-1 to E-cadherin expression [29]. Further, another group demonstrated a direct association between SNAIL-1 and Cadherins [29]. Recently, Shin et al demonstrated that over-expression of SNAIL-1 significantly enhanced tumor progression, lymphovascular invasion, lymph node metastases and perineural invasion [30].

Earlier studies by Gottwein et al showed that Herpesviruses can inhibit p21 expression and attenuates p21-mediated cell cycle arrest [31]. Furthermore, a study from Takahashi et al also suggested that SNAIL represses p21 expression in the process of cellular differentiation [32]. Previous studies have also suggested that NF-kB signaling is important in KSHV-mediated oncogenesis [33,34] and the family of matrix metalloproteinase (MMPs) (zinc-dependent photolytic enzymes) are involved in many physiological and pathological events associated with the virus [35]. It is also known that numerous modulatory processes are regulated by MMPs to drive malignant progression of cancers. These include induction of cell invasion, release of growth factors, remodeling of ECM, promotion of angiogenesis, or modulation of the local immune responses [36]. Importantly MMP9 a well-studied MMP that induces cell invasion and metastasis in various cancers [37], was shown to be induced by the EBV oncoprotein, LMP1 [38]. Therefore understanding how these EMT markers are influenced in KSHV-mediated oncogenesis, and specifically regulated through one of the essential viral-encoded latent antigen LANA will provide important clues as to their contribution to viral-associated pathologies.

## Results

### KSHV infection leads to Par3 up-regulation

**Fig 1:**
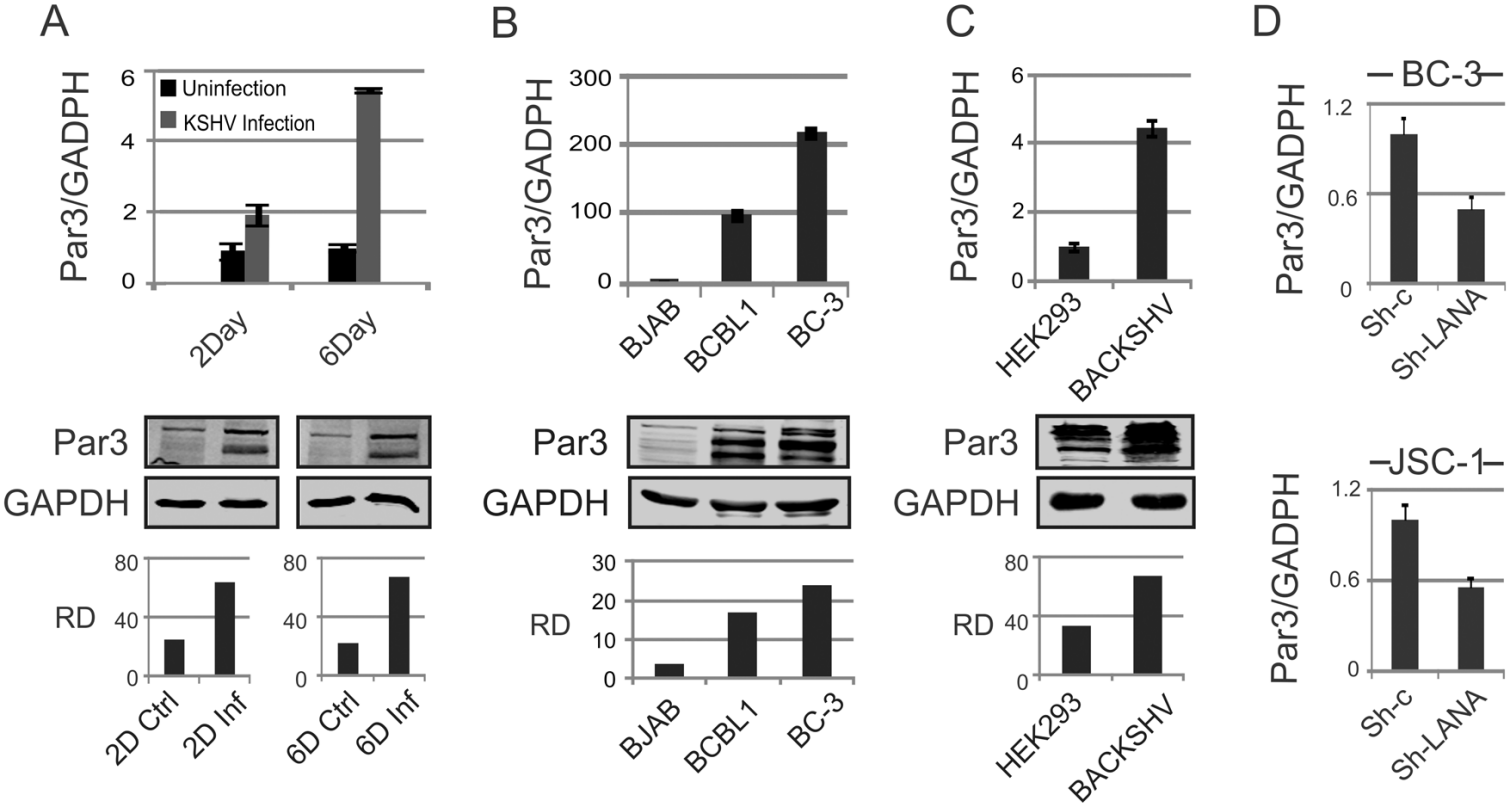
KSHV infection leads to Par3 up-regulation. (A) Expression of Par3 was examined at the transcript and protein levels by real-time PCR and Western blot in KSHV infected PBMCs at day 2 and 6. Here RQ and RD terms are using for relative quantification and relative density, respectively. (B) Par3 were measured at the transcript and protein levels in BJAB (KSHV negative) and BCBL1 and BC-3 (KSHV positive) cell lines. (C) Par3 levels were measured in HEK-293 and HEK-293-BACKSHV at the transcript and protein levels. (D) KSHV positive cells (BC-3 and JSC-1) were knockdown with LANA and compared with controls to assessed transcript of Par3.

Earlier studies from our group investigating KSHV infection of primary blood mononuclear cells (PBMCs) identified a number of genes related to DNA damage and regulators of virus infection [39]. One gene is particular was dramatically changed after KSHV infection. Surprisingly this enhancement was distinctly different from other previously reported genes shown to be important for KSHV-induced oncogenesis. More specifically, we found that infection with KSHV led to an increase of Par3 levels at day 2 and 6 in KSHV infected primary B-cells as seen at the transcript as well as protein levels (Fig 1A). Additionally, we also obtained a similar pattern for Par3 expression when we looked at KSHV positive cells compared to KSHV negative cells at both the transcript and protein levels (Fig 1B). We further compared mock HEK-293 cells with stable HEK-293-BAC-KSHV cells selected with hygromycin. As expected, we found consistent up-regulation of Par3 in the presence of KSHV (Fig 1C). These results strongly suggest that Par3 is strongly up-regulated by KSHV and may likely play a key role in viral-mediated cell transformation. Earlier studies have shown the importance of LANA in KSHV infection [23,40,41,42,43,44]. To identify the KSHV-encoded antigen responsible for upregulation of Par3, we analyzed the knock-down of LANA in KSHV-positive BC-3 and JSC-1 PEL cell lines. Transcription analysis by quantitative Real-Time PCR showed a consistent drop of greater than 50% in Par3 transcript levels in BC-3 and JSC-1 cell lines stably knocked down for LANA using a Lentivirus shRNA to target LANA (sh-LANA) (Fig 1D).

### LANA interacts and co-localizes with Par3

**Fig 2:**
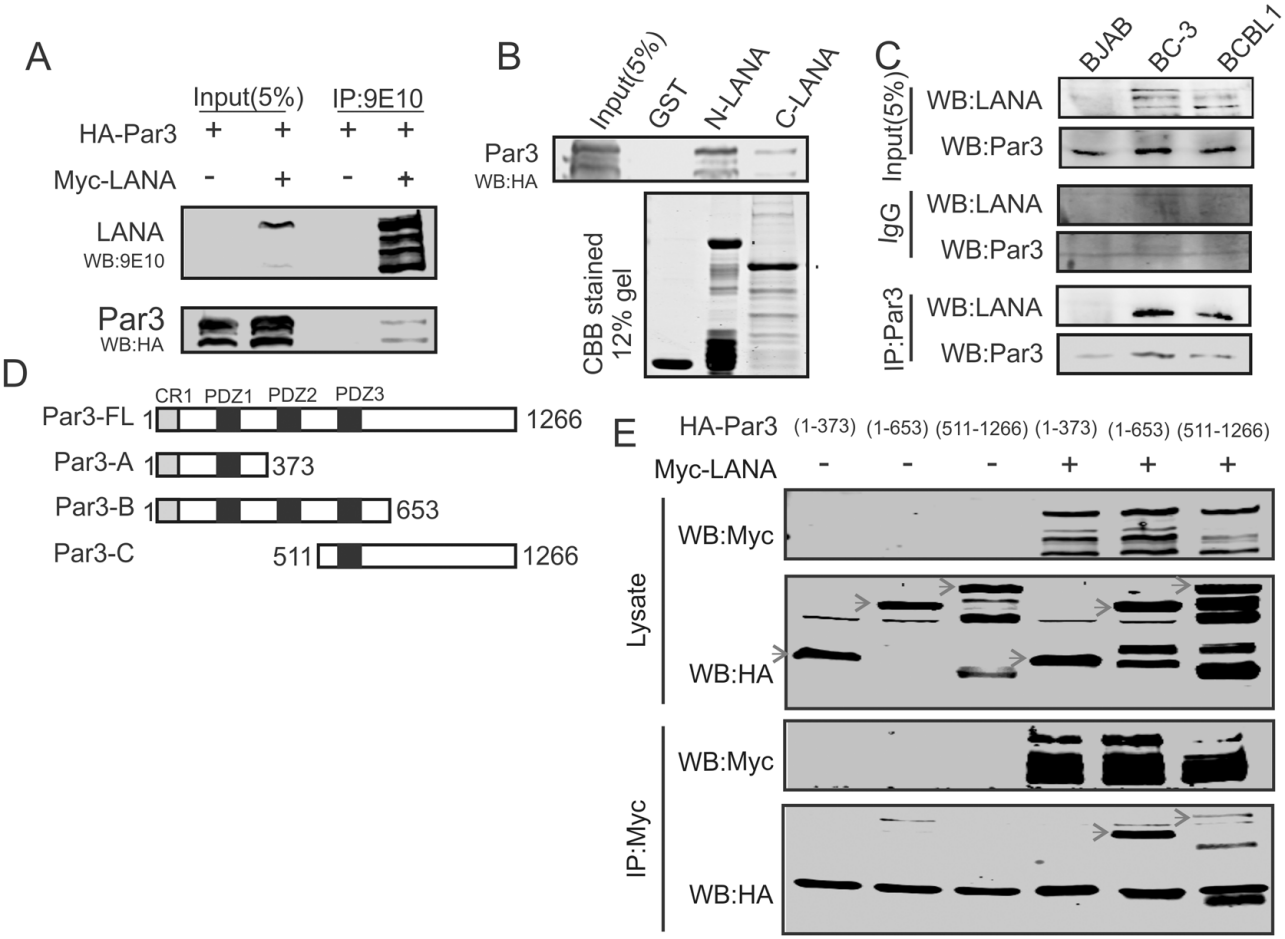
LANA interacts and colocalizes with Par3. (A) Co-Immunoprecipitation of Par3 with LANA was examined in HEK-293 cells. Myc tagged LANA and HA tagged Par3 constructs were used in this experiment. (B) GST pull-down assays shows binding of Par3 with N- (1–340 aa) and C-terminus (930–1162 aa) of LANA. GST-N-LANA and GST-C-LANA bound to Glutathione Sepharose beads were used to pull down Par3 from HA-Par3 transfected HEK-293 cell lysates. (C) Endogenous immunoprecipitation assays with Par3 antibody in KSHV negative BJAB and KSHV positive (BC-3 and BCBL1) cell line was performed. (D) Par3 domains and truncations used for mapping the interaction domain. (E) Co-Immunoprecipitation of Par3 truncations (1–1266, 1–373, 1–653 and 511–1266 aa) with LANA was examined in HEK-293 cells.

**Fig 3:**
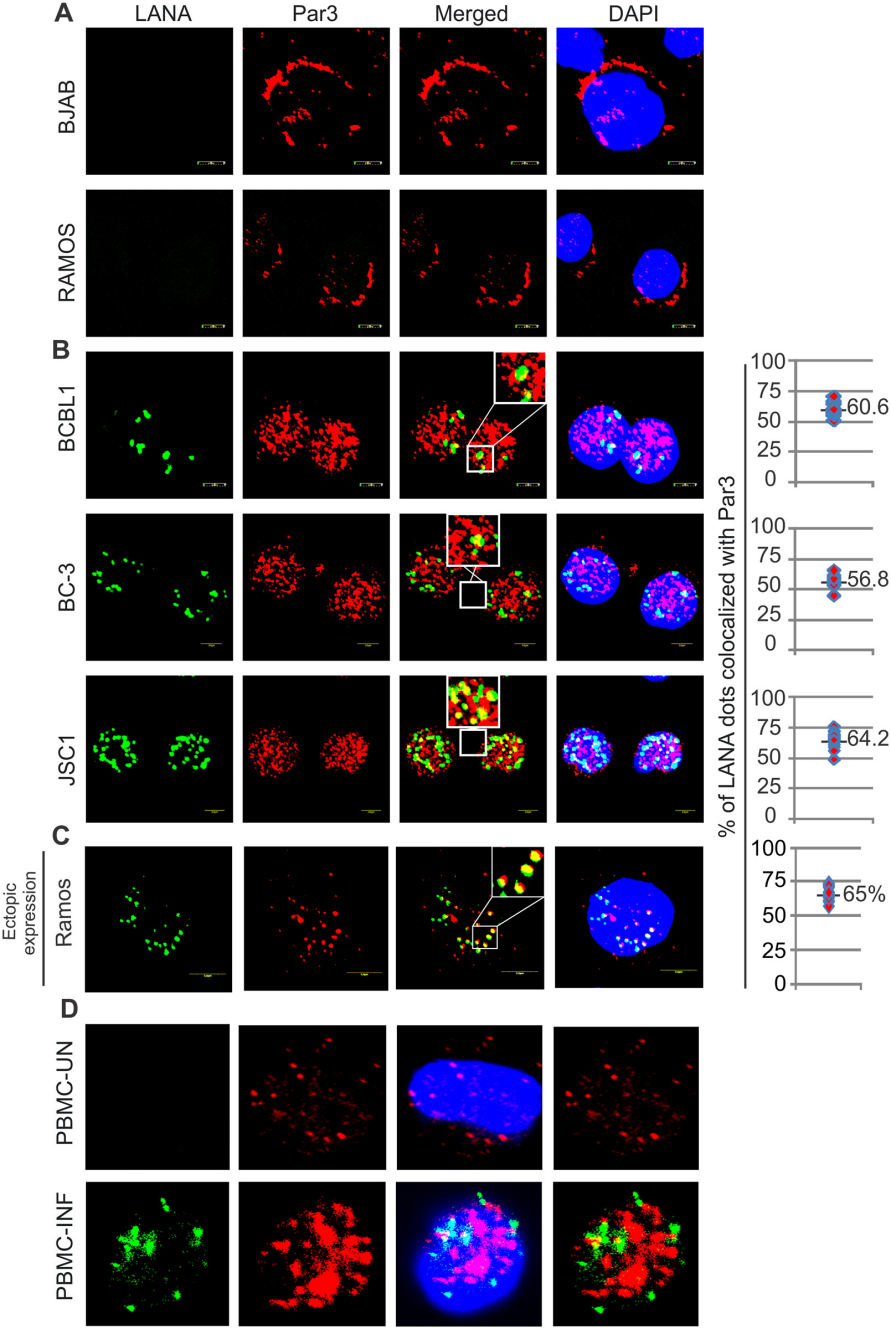
KSHV infection leads to Par3 nuclear localization. (A) KSHV negative BJAB, Ramos, (B) KSHV positive BCBL1, BC-3, JSC-1 cell lines and (D) PBMCs uninfected and infected with KSHV at day 6 were used to determine the localization of Par3. LANA staining was used as positive control. In KSHV positive cells graphs were plotted with the percent of LANA foci colocalized with Par3. (C) Co-localization of LANA with Par3 was performed in KSHV negative Ramos cell line. The graphs represent the percent of LANA foci co-localized with Par3.

To investigate whether the regulation of Par3 in KSHV-positive cells is directly linked to LANA expression, we asked whether LANA can associate in a complex with Par3. In HEK-293 cells using ectopic expression, we demonstrated that Par3 was present in a complex with LANA (Fig 2A). A number of earlier studies showed that LANA is a major transcription factor which is an important contributor to KSHV-mediated oncogenesis [40,41,43,44,45]. We validated our immunoprecipitation results by exploring this with an *in-vitro* approach. We used GST pull down assays and in support of the results above, we found that an N-terminal domain of LANA had a stronger binding activity when compared to a C-terminal domain (Fig 2B). To further support our interaction results between LANA with Par3, we performed endogenous immunoprecipitation assays using Par3 specific polyclonal antibody with the appropriate isotype control in two KSHV-positive PEL cell lines BC-3 and BCBL1 (Fig 2C). BC-3 and BCBL1 cells showed a stronger signal for Par3 which was also able to specifically immunoprecipitate LANA in Par3 pull down complexes from whole cell lysates. To more closely examine the domain of Par3 responsible for interaction with LANA we generated truncations of Par3 described earlier (Fig 2D) [46], and performed immunoprecipitation assays (Fig 2E). Here we show that residues 373 to 653 of Par3 which contains PDZ domains 2 and 3 bound very strongly with LANA when compared to other Par3 regions (Fig 2E). To further support our association studies between LANA and Par3, we used confocal microscopy to determine co-localization of these two proteins in the B-cell line Ramos (Fig 3C). The results showed that LANA and Par3 colocalized greater than 60% in these assays in the nuclear compartment (Fig 3C). We have also seen that Par3 localization was predominantly in the periphery on the outside of the cytoplasm and cell membrane in the absence of LANA (S1 Fig). Some signals were seen in the nucleus at a lesser extent (S1 Fig).

### LANA can induce nuclear translocation of Par3

Previous studies localized Par3 signals along the periphery of the cell membrane [41]. However, some Par3 signals were also seen in the nucleus suggesting signaling activities that modulate specific cellular processes at the cell membrane as well as in the nucleus [47]. In this study we were interested in determining the localization of Par3 during KSHV infection and more specifically in the presence of LANA. Par3 staining was observed in KSHV negative cells (BJAB and Ramos) to be mostly at the periphery of the cells compared to a shift of the majority of Par3 signals to the nuclear compartment in KSHV-positive cells (BC-3, BCBL1 and JSC-1) (Fig 3A and 3B). Moreover, changes in localization of Par3 in the presence of KSHV were consistent across different cell lines although these cell lines may have different genetic backgrounds. Interestingly, around 60% of nuclear Par3 signals was colocalized with LANA although most of the signals were nuclear (Fig 3B). Additionally, in PBMCs infected with KSHV the localization pattern of Par3 changed predominantly to nuclear compared to control PBMCs on day six (Fig 3D). Importantly, the expression of Par3 was also significantly enhanced on KSHV infection when compared to uninfected PBMCs (Fig 3D).

### KSHV-encoded LANA stabilizes Par3

**Fig 4:**
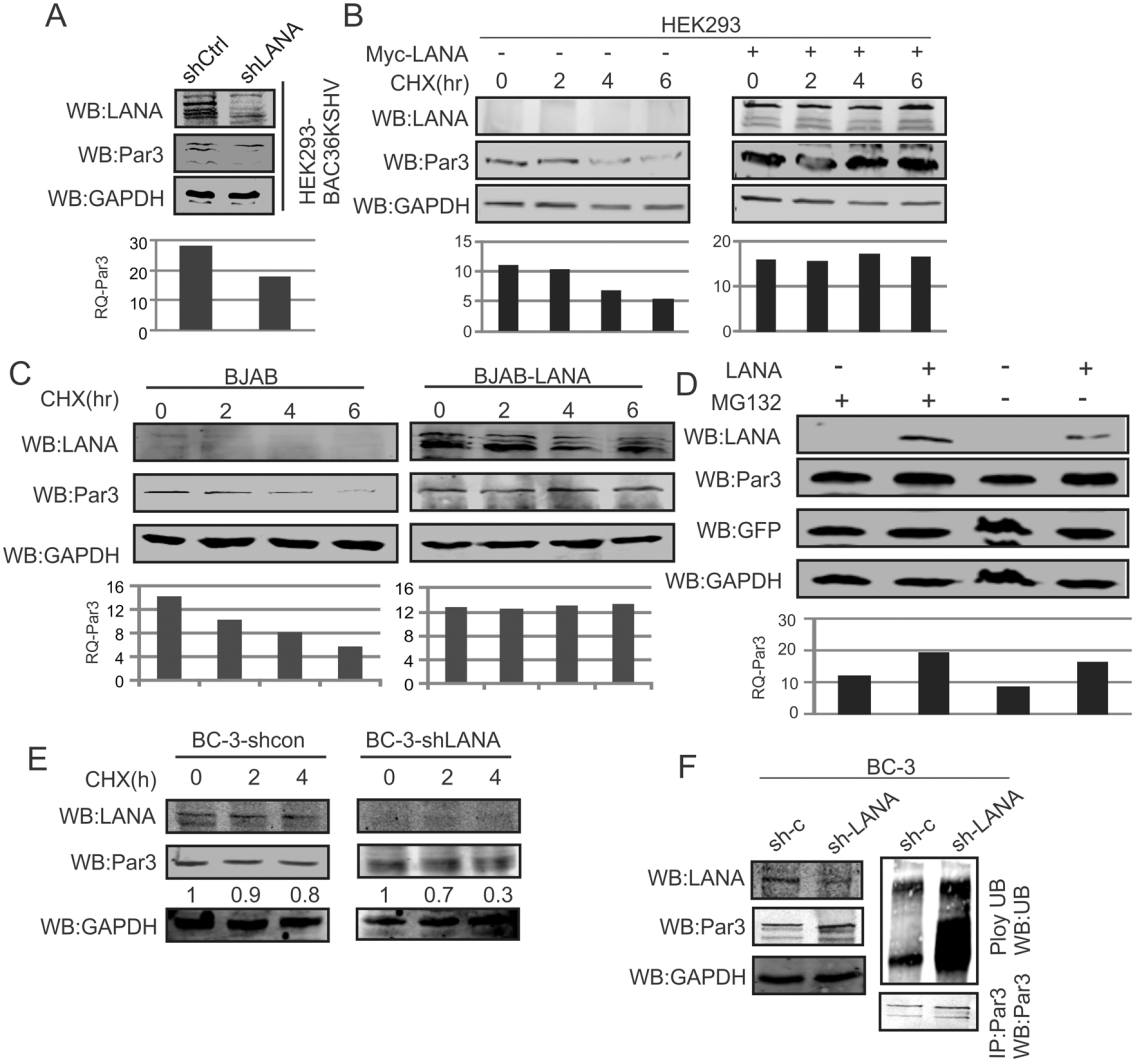
LANA stabilizes Par3 in KSHV positive cells. (A) HEK-293-BAC-KSHV cells were transfected with sh-LANA and sh-Control. Endogenous Par3 expression was measured and presented in graph. GAPDH was used as a protein loading control. (B, C) Stabilization of Par3 was examined with cyclohexamide in HEK-293 and BJAB cells. Left panels and right panels were used as vector control and LANA expression, in a time dependent manner. (D) The proteosome inhibitor MG132 was used to determine if Par3 stability was linked to the proteosome degradation pathway. GFP and GAPDH were used as transfection and endogenous protein loading controls. (E) BC-3-shControl and BC-3-shLANA cells were treated with cyclohexamide and observed Par3 endogenous on hours dependent manner. (F) BC-3-shControl and BC-3shLANA cells were treated with MG132 and followed immunoprecipitation of Par3. Endogenous ubiquitin and Par3 were detected using specific antibodies in both the cell lines. GAPDH was monitored as an internal control for loading in the input section.

Identifying the strong association of LANA with Par3 indicates a possible functional role for this complex. Therefore we evaluated whether LANA contributed to increased Par3 levels, as well as determining whether an increasing amount of LANA had any significant effects on the expression or stability of Par3. Here HEK-293-BAC-KSHV cells were transfected with sh-LANA to knockdown LANA and a control knockdown vector containing a scrambled sequence as control (Fig 4A). The results demonstrated that LANA played a role as a crucial regulator of Par3 and may be important for KSHV-induced oncogenesis. Further we evaluated the stability of Par3 in the absence or presence of LANA (Fig 4B and 4C). Interestingly, we observed that LANA significantly stabilizes Par3 and was consistent in HEK-293 as well as BJAB cells, a B-cell line (Fig 4B and 4C). However, the mechanism utilized by LANA for the stabilization of Par3 was not previously explored. To address this question we treated cells with the proteasome inhibitor MG132 in the presence or absence of LANA compared to a DMSO control in BJAB cells. The results showed that as expected, Par3 was stabilized in the presence of LANA (Fig 4D). We further performed cyclohexamide experiments in BC-3-shControl and BC-3-shLANA cell lines to block *de novo* protein synthesis. A more rapid reduction in Par3 levels were seen in LANA-negative sh-LANA BC-3 cells compared to BC-3 with the sh-RNA control (Fig 4E). To further confirm our results in LANA knockdown of BC-3, we utilized MG132 in BC-3 sh-Control and sh-LANA with pull down of Par3. As expected, we observed greater degradation of Par3 in LANA knockdown compared to the control BC-3 cell background (Fig 4F). This strongly suggested that in KSHV positive cells LANA is a major contributor to stabilization of Par3.

### Par3 knockdown leads to delay in cell proliferation

**Fig 5:**
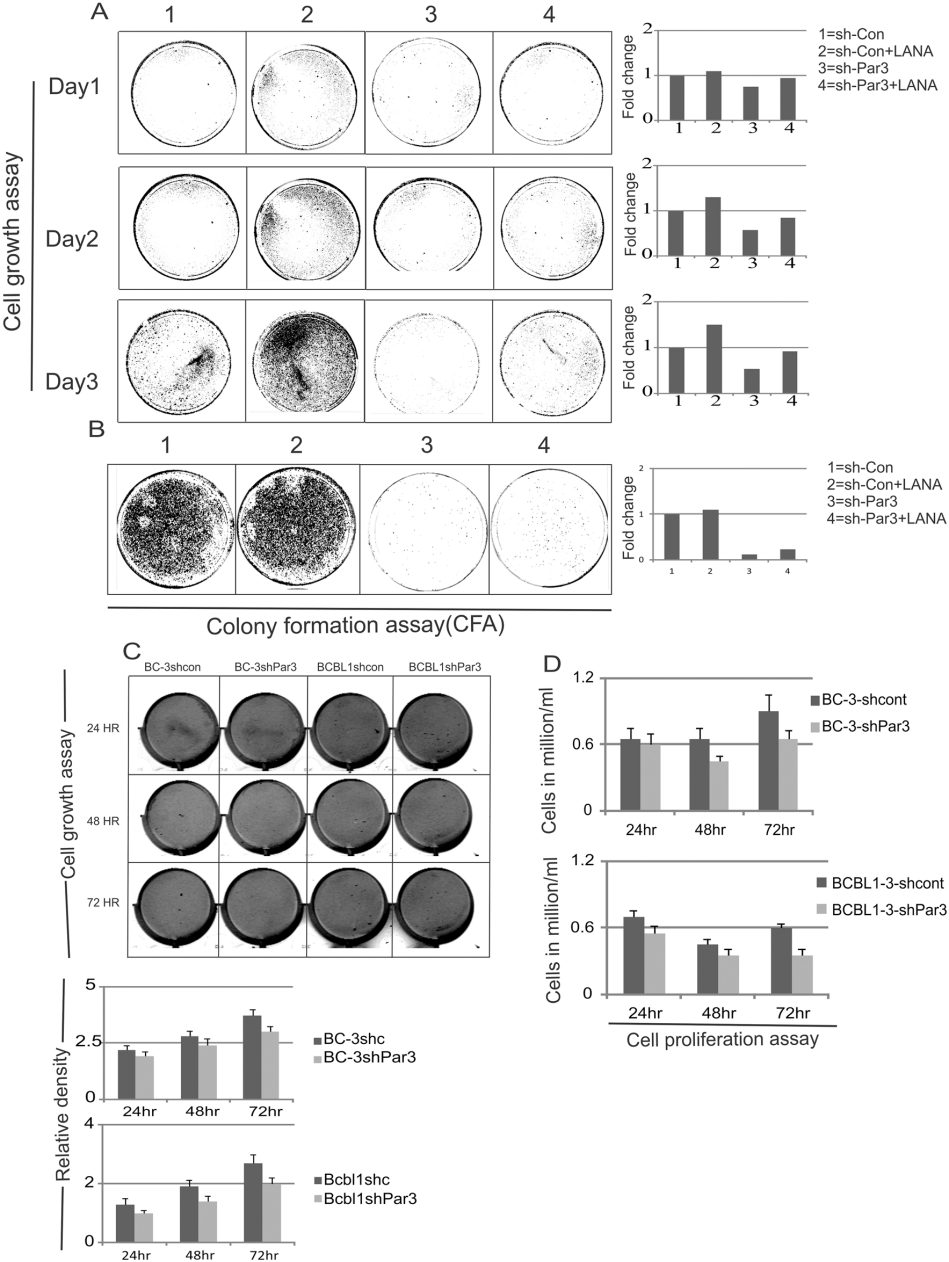
Par3 knockdown leads to delay in cell proliferation. (A) Cell growth assay was carried out in HEK-293 cells. As indicated plasmids were transiently transfected in corresponding plates. 48 hour post transfection, 50,000 cells were plated for all groups into 100 mm dishes. Further cell density was scanned after fixing with 3% PFA and staining with crystal violet. Graphs are presented on the basis of intensity of colonies. Li-Cor Odyssey Scanner was used for scanning these plates. (B) Colony formation assays were carried out under puromycin and G418 antibiotic selection using HEK-293 cell lines. Representative graphs were also plotted for every set of experiments. Quantitation was done on the basis of intensity of colonies in every plates. (C) Cell growth assays were carried out in BC-3 and BCBL1 cells. As indicated plasmids were transiently transfected and transferred to corresponding flasks. 48 hour post transfection, 50,000 cells were plated for all groups into 6 well plates. Further cell densities were determined. Graphs are presented on the basis of intensity of cells. Li-Cor Odyssey Scanner was used for scanning the plates. Plotted graph based on cell density. (D) Cell proliferation assays were performed by using Trypan blue staining. 48 hour transfection, cells were counted for day1, 2 and 3 and plotted for live cells accordingly.

Our studies so far showed that Par3 was up-regulated and stabilized by LANA. Further, Par3 interacted and was predominantly translocated to the nucleus by LANA. Furthermore, we were interested in determining the contribution of Par3 to cell proliferation. Here we used cell growth assays (Fig 5A and 5C), colony formation assays (Fig 5B) and cell number determination to evaluate cell proliferation (Fig 5D). We used Par3 knockdown cells compared to the scrambled controls in HEK-293 and BC-3, BCBL1 cells (Fig 5A and 5C, S2A Fig respectively). The results showed a moderate inhibition of cell growth and proliferation with knockdown of Par3 when compared to the vector control cell lines (Fig 5A and 5C, S2A Fig). Interestingly, we observed that in the presence of LANA no induction in cell proliferation in the Par3 knockdown cells was observed when compared to control cells (Fig 5A). To quantify the proliferation activity we performed cell counting assays for up to 6 days. Cells were selected with puromycin after 24 hour post transfection and plotted for cell density (S2B Fig). Par3 knockdown cells showed a significant drop in growth capabilities compared to the corresponding control cell lines (S2B Fig). We also showed that the efficiency of Par3 knockdown achieved at the transcript level was greater than 80% (S2C Fig). Importantly, Par3 knockdown led to retarded growth patterns in HEK-293 as well as BC-3 and BCBL1 which ranged from 30–75% (Fig 5B and 5D, S2A and S2B Fig respectively). In the colony formation assays, we demonstrated that Par3 knockdown resulted in more than a 80% drop in colonies compared to controls (Fig 5B). Similar to our cell growth assays, the colony formation assays showed a significantly higher number of colonies with LANA in the Par3 knockdown cells (Fig 5B). In our cell growth assays we measured cell density in BC-3 and BCBL1. There were no dramatic changes. This is likely due to the inclusion of dead cell density in the total cell density as they were not washed off before image processing. However, when counting live cells with Trypan blue the changes were dramatic and Par3 knockdown led to slower cell proliferation (Fig 5C and 5D). This strongly suggested an oncogenic property of Par3 which is directly regulated by LANA and contributes to progression of KSHV-induced cell proliferation. Additionally, de-regulation of apoptosis by Par3 may also be an important contributor to the control of cell growth and proliferation. Previous studies have demonstrated that genes with oncogenic properties also have anti-apoptotic activity [48,49,50]. Here we also observed that knockdown of Par3 led to increased apoptotic activity when monitored by serum starvation or etoposide treatment (S2D and S2E Fig). Induction of apoptosis was much higher (15 fold) in Par3 knockdown compared to vector control (3 fold) in serum starve cells (S2F Fig). Similarly, etoposide treatment led to an increase by 13 fold in cell death for Par3 knockdown cells compared to control vector (S2F Fig). These results strongly demonstrated that knockdown of Par3 enhanced the apoptotic activities induced by serum starvation or etoposide as seen by an increase in cell death (S2A–S2F Fig). This strengthens our hypothesis that Par3 has an oncogenic role in KSHV-infected cells. Par3 knockdown and exogenous expression of LANA were confirmed through quantitative real time PCR (S3A–S3C Fig).

### Status of EMT markers in KSHV infected PBMCs and Par3 knockdown cells

**Fig 6:**
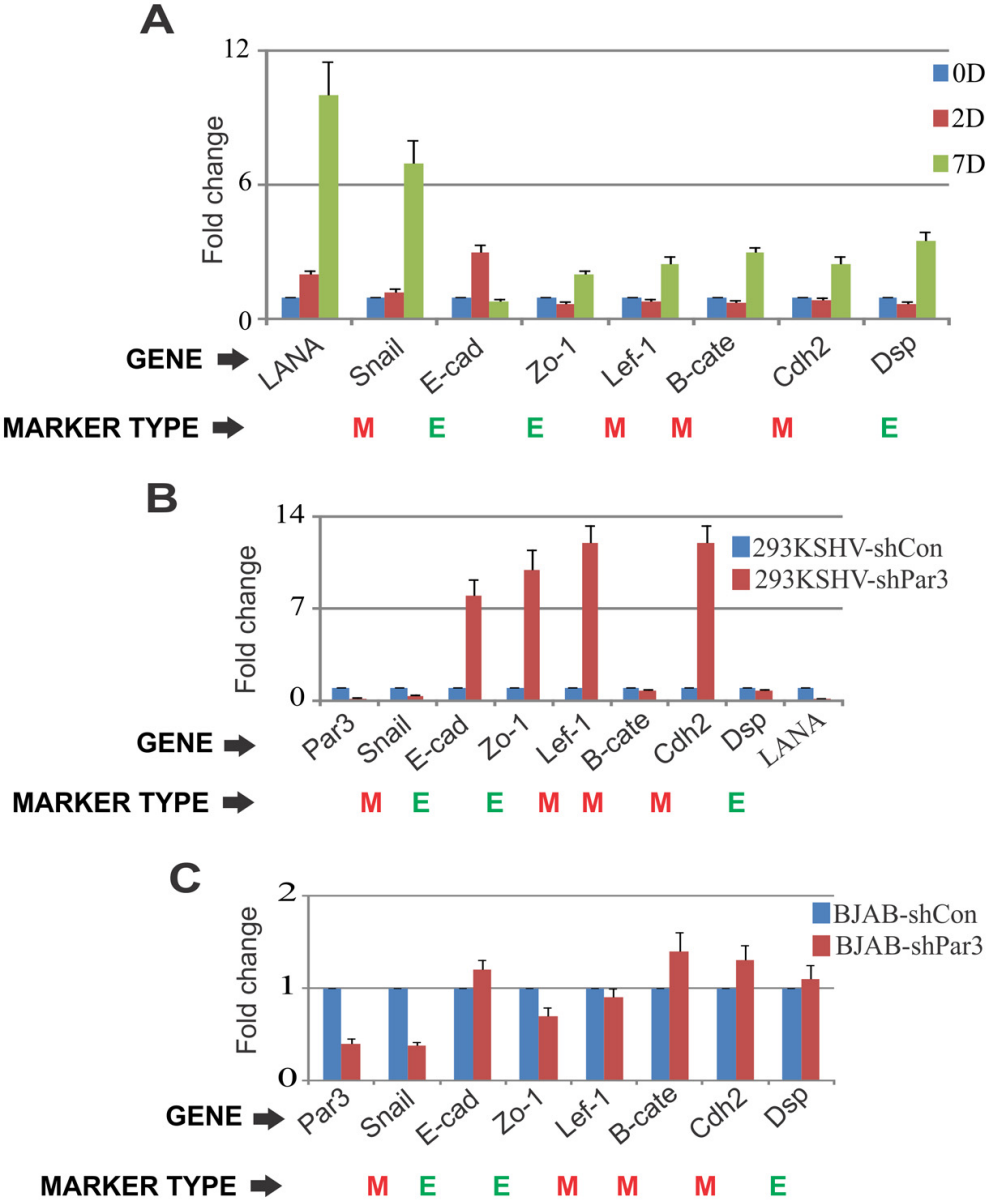
Status of EMTs in KSHV infected PBMCs and Par3 knockdown cells. (A) PBMCs were subjected to infection with KSHV and analyzed using days post-infection for the screening of epithelial (E-cadherin, Zo-1, Dsp) and mesenchymal (Snail, Lef1, B-catenin, Cdh2). qRT-PCR was performed for the transcript analysis of EMTs (epithelial to mesenchymal markers). (B) HEK293-KSHV-shControl and HEK-293KSHV-shPar3 cells were used to study these EMTs. qRT-PCR was performed to determine the fold changes for EMTs and to confirm the Par3 knockdown in HEK-293KSHV stable cell lines. (C) BJAB-shContol and BJAB-shPar3 cells were generated to study these EMTs. qRT-PCR was performed to analyze the fold changes for EMTs and to confirm Par3 expression in transiently transfected BJAB cells.

How KSHV-induces EMT markers in B-cell lymphoma have not been previously explored. Par3 was previously described as an important factor for cells to transition from epithelial to mesenchymal [51]. However, this has not been previously explored in B-cell lymphoma. To examine its role in KSHV infected PBMCs, we screened a number of epithelial (E-cadherin, Zo-1, DSP) and mesenchymal (SNAIL, Lef1, B-catenin, Cdh2) markers in KSHV infected PBMCs at day 2 and 7 (Fig 6A). Surprisingly, we showed that SNAIL expression was higher compared to other EMT markers (Fig 6A). Here LANA was used as a positive control for KSHV infection (Fig 6A). Further we wanted to explore the interference of Par3 in the presence of KSHV infection. Hence, we evaluated EMT markers in HEK293 stably expressing the BAC-KSHV with shControl, -shPar3 (Fig 6B). Interestingly, we showed that Par3 knockdown led to a significant downregulation of SNAIL (Fig 6B). Additionally, we observed that E-cadherin and Zo-1 levels were elevated in Par3 knockdown cells (Fig 6B). Moreover, Par3 knockdown in BJAB cells led to SNAIL downregulation and E-cadherin upregulation albeit moderately (Fig 6C). These results indicated that SNAIL was positively regulated in KSHV infection through Par3, and that E-cadherin was negatively regulated when compared to the other EMT markers (Fig 6A–6C).

### Epithelial to mesenchymal cell transition markers are modulated by SNAIL and Par3 expression in KSHV infected B-cells

**Fig 7:**
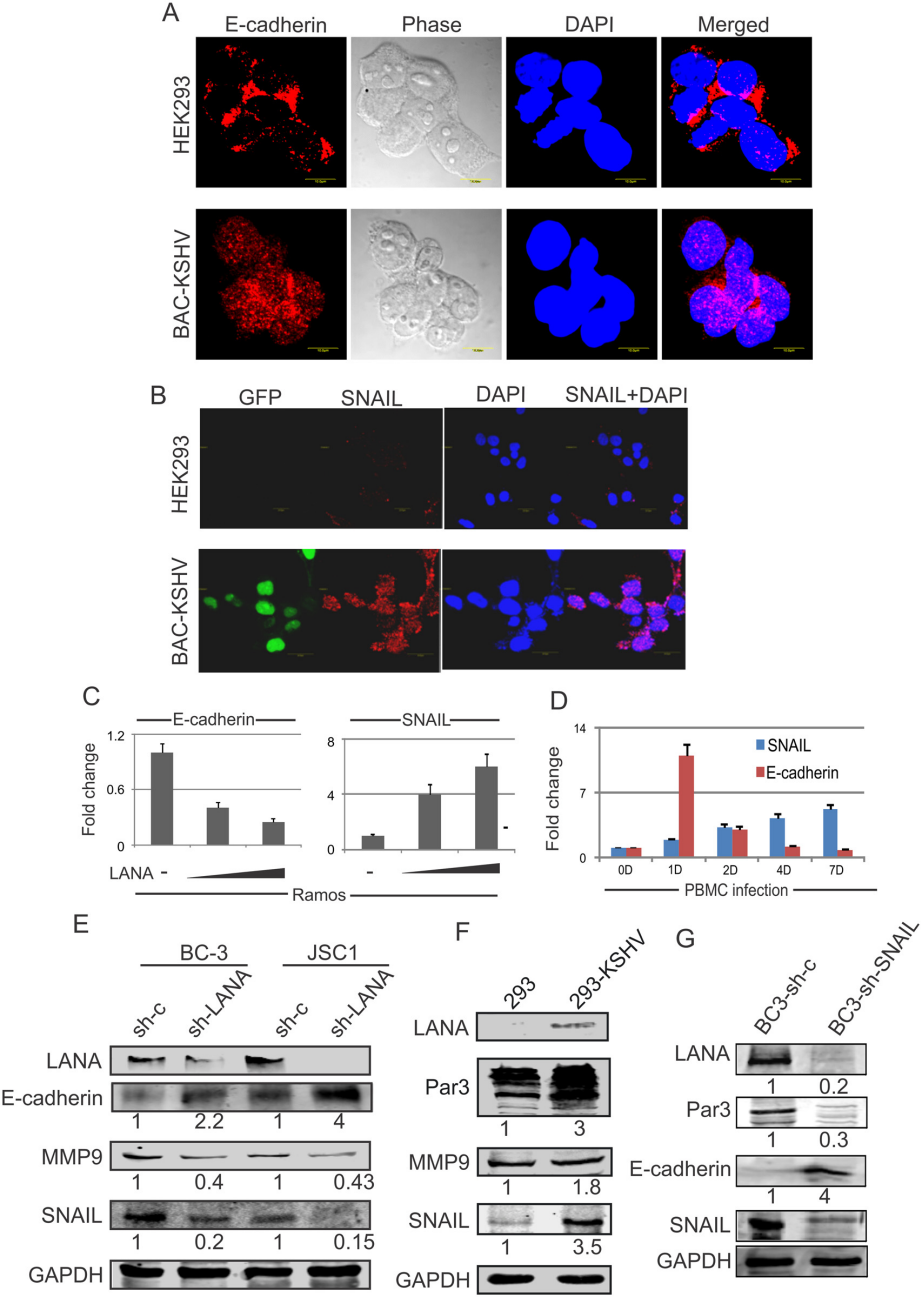
KSHV can induce epithelial to mesenchymal transition markers in infected B-cells. (A, B) HEK-293 and BAC-KHSV cells were probed with the antibodies against the EMT markers E-cadherin, and SNAIL. DAPI was used to stain the nucleus. (C) Ramos cells were transfected with an increasing dose of LANA to evaluate the transcript levels of E-cadherin and SNAIL. (D) KSHV infection was carried out in primary PBMCs and monitored up to 7 days post-infection. Control cells without KSHV infection was measured for days 1, 2, 4 and 7 for the measurement of SNAIL and E-cadherin transcripts. (E) Stable LANA knockdown cells were compared to controls for the measurement of Par3, E-cadherin, MMP9 and SNAIL. LANA blots were used as a positive control to monitor its expression in cells, and GAPDH was used as a protein loading control. (F) HEK-293-BAC-KSHV compared to HEK293 cells were evaluated at the protein levels for the expression of Par3, E-cadherin, MMP9 and SNAIL. LANA blots were used as a positive control for the expression of cells and GAPDH was used as a protein loading control. (G) SNAIL knockdown in BC-3 cells were evaluated with control vector for the measurement of the expression of LANA, Par3, and E-cadherin. GAPDH was used as a protein loading control.

Based on the above data we monitored the localization or expression pattern of E-cadherin and SNAIL in HEK-293 cells and BAC-KSHV infected cells (Fig 6A–6C). Surprisingly in the presence of KSHV, these EMTs markers were translocated predominantly to the nucleus from the periphery of the infected cells (Fig 7A and 7B). These results clearly indicate a potential mechanism by which these EMTs markers may be regulated by KSHV encoded antigens after infection. We also observed enhanced signals for E-cadherin in HEK-293 compared to BAC-KSHV infected cells and conversely, SNAIL signals were much stronger in BAC-KSHV infected cells compared to HEK-293 control cells. To corroborate our findings, we performed Western blot analysis for E-cadherin, SNAIL, and MMP9 as well as LANA and Par3. Importantly, the levels of MMP9, SNAIL and Par3 were up-regulated in BAC-KSHV infected cells compared to control cells (Fig 7F). These results establish that Par3, E-cadherin, SNAIL and MMP9 are contributors to KSHV-mediated activities in the B-cell background.

Previous studies suggested that SNAIL and E-cadherin had opposing roles during EMTs leading to metastasis [29]. In follow-up to this result, we determined the transcript levels of SNAIL and E-cadherin in the B-cell line Ramos expressing increasing amounts of LANA. Here we also found that SNAIL was up-regulated and E-cadherin was down-regulated as the amount of LANA increased (Fig 7C). In the above studies we demonstrated that KSHV infection of PBMCs at day 2 and 7 modulated EMT markers. To more closely monitor the expression patterns in KSHV-infected PBMCs, we examined these changes at days 1, 2, 4 and 7 (Fig 7D) specifically for SNAIL and E-cadherin transcripts (Fig 7D). As expected, we showed that E-cadherin expression was significantly induced at day 1 followed by down-regulation through day 7. Notably, SNAIL expression was consistently upregulated from day 1 to 7 (Fig 7D), which strengthens our hypothesis that SNAIL and E-cadherin plays an important role in KSHV infection. To determine whether these markers are directly linked to LANA expression, we evaluated the expression of SNAIL, Par3, E-cadherin and MMP9 in LANA knockdown KSHV-positive BC-3 and JSC-1 cell lines (Fig 7E). Importantly, SNAIL levels was reduced in LANA knockdown KSHV positive cells and so provided another clue as to the mechanism by which SNAIL can be regulated by LANA during KSHV infection (Fig 7E). The levels of E-cadherin was strongly upregulated in the LANA knockdown cell lines and the level of increase inversely correlated with the degree of LANA knockdown (Fig 7E). The degree of SNAIL suppression was also directly related to the level of LANA knockdown (Fig 7E). Importantly, MMP-9 another EMT marker, was also reduced in LANA knockdown cells (Fig 7E). Similarly, changes in levels of MMP-9, E-cadherin and SNAIL were seen with infection of HEK-293 cells with BAC-KSHV infection (Fig 7F). Moreover, knockdown of SNAIL in BC-3 compared to a control shRNA showed a dramatic reduction of LANA and Par3 while E-cadherin was upregulated in SNAIL knockdown cells (Fig 7G). These results strongly supported a positive association of LANA with Par3 and SNAIL as well as a dramatic loss of E-cadherin expression in KSHV infected cells.

### Par3 and SNAIL expression were enhanced in KSHV induced tumors in a NOD/SCID xenograft model

**Fig 8:**
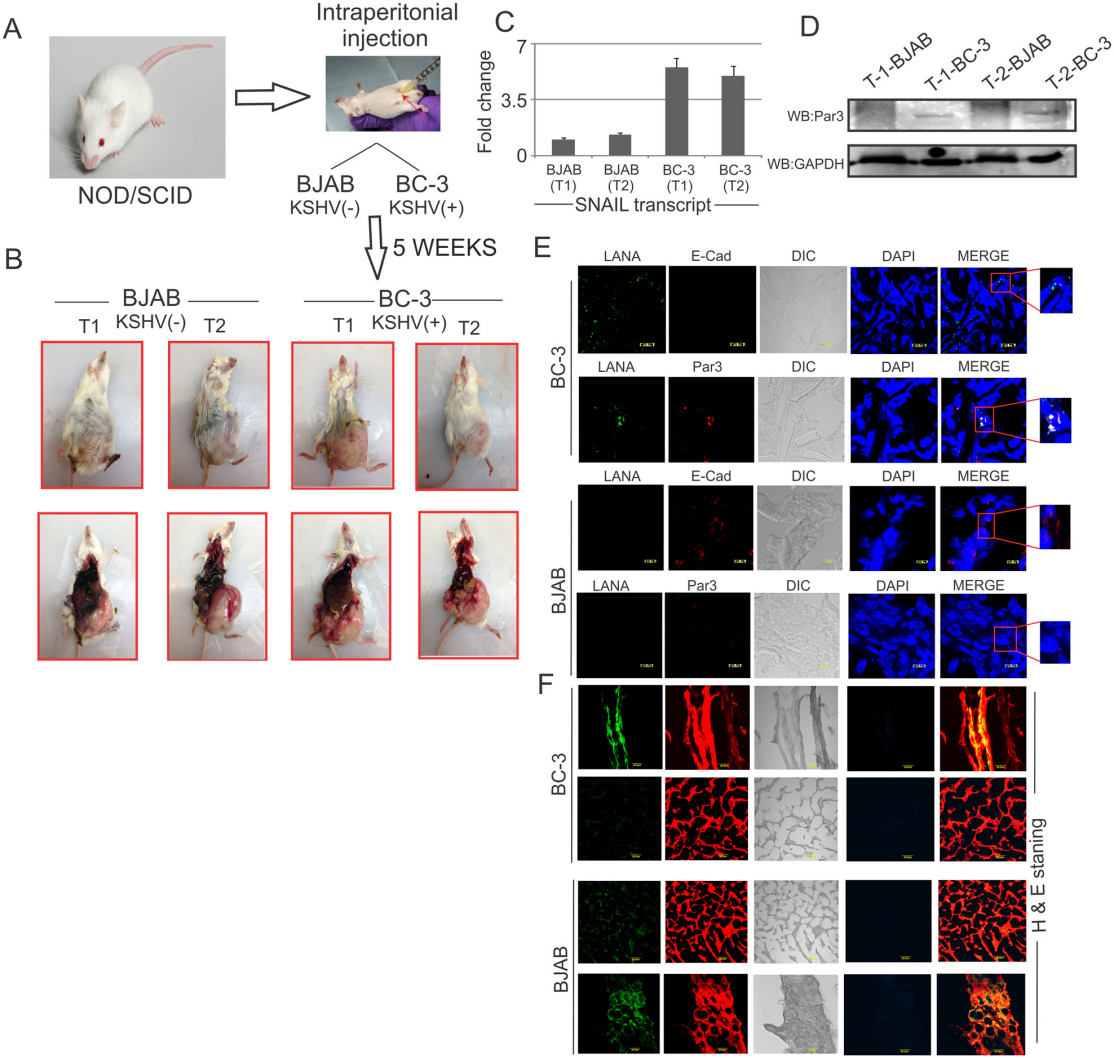
Expression of SNAIL and Par3 in BJAB and BC-3 tumors in mouse xenograft model. (A, B) NOD/SCID mice were injected with ten millions of BJAB and BC-3 cells intraperitoneal. (C, D). After 5 weeks, the mice were scarified and tumor dissected for analysis of transcripts and proteins of SNAIL and Par3 in these tumors. (E) Immunohistochemistry were performed on BJAB and BC-3 generated tumors. Here we used primary antibodies against LANA, E-cadherin and Par3. DAPI was used for nuclear staining. (F) H and E staining was shown for a similar group of tumor tissues capitalize to Fig 8F.

To determine if our *in vitro* findings for Par3 and SNAIL expression correlated with that seen *in vivo*, we generated tumors in a NOD/SCID mouse xenograft model with KSHV positive BC-3 and KSHV negative BJAB cells (Fig 8A). Ten million cells were injected intraperitoneally. Mice were sacrificed approximately 5 weeks after injection with BJAB and BC-3 cells (Fig 8B). Further, the tumors were harvested and subjected to transcript and Western blot analyses (Fig 8C and 8D). SNAIL transcripts were substantially upregulated in BC-3 generated tumors compared to BJAB. Similarly, Par3 protein expression was observed more prominently in BC-3 generated tumors compared to BJAB. We also performed IHC in these tumors with primary antibodies against LANA, E-cadherin, Par3, and DAPI for nuclear staining (Fig 8E). Here we saw enhanced staining for Par3 and LANA in BC-3 induced tumors. However, E-cadherin staining was prominent in BJAB induced tumors but not in KSHV positive BC-3 cells (Fig 8E). H and E staining was shown for a similar group of tumor tissues capitalize to Fig 8F. Overall, these results clearly showed enhanced expression of Par3 and SNAIL in KSHV positive tumors compared to KSHV negative tumors.

### SNAIL bound to the p21 promoter in KSHV positive cells

**Fig 9:**
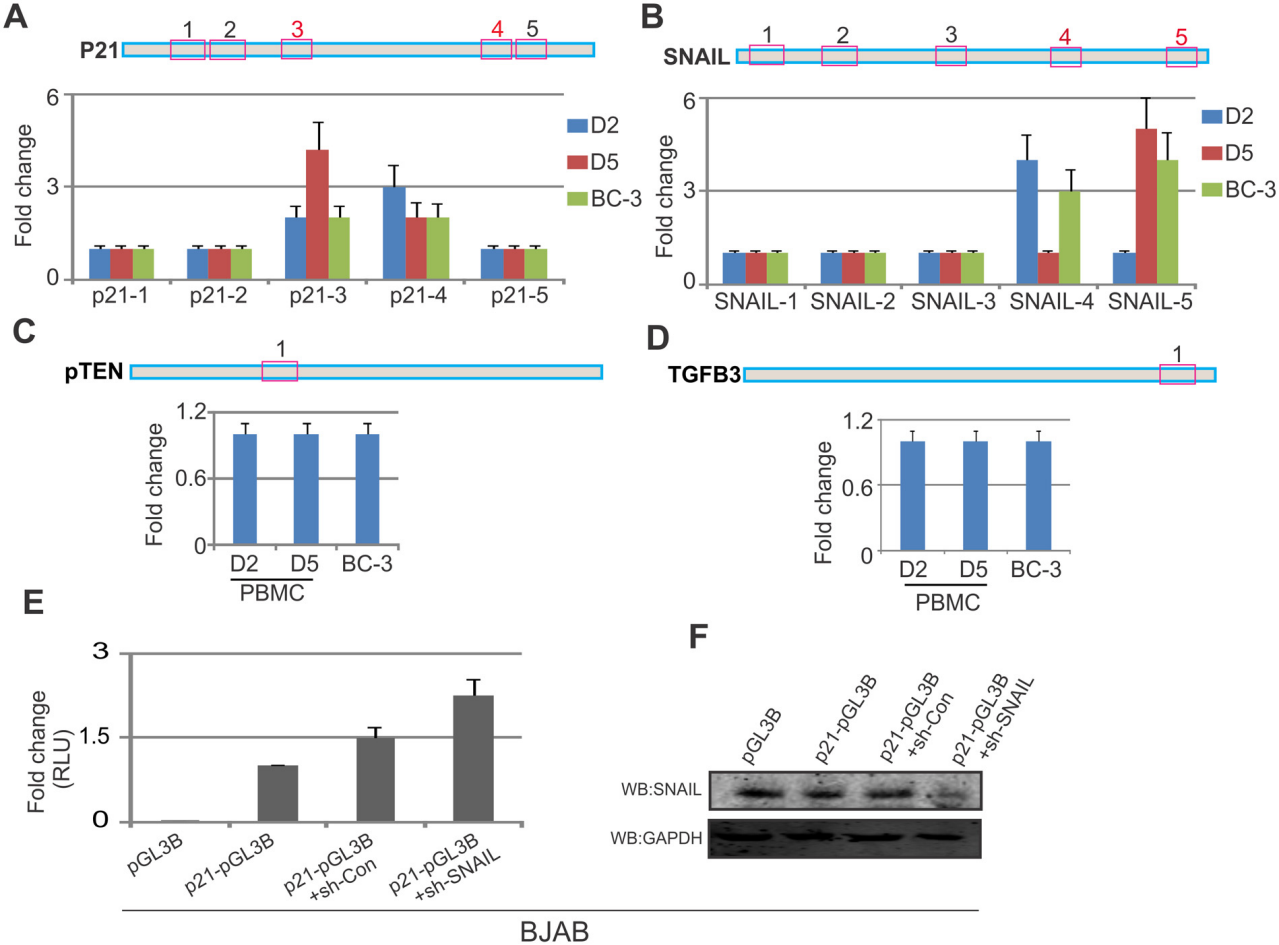
SNAIL can transcriptionally repress p21 in KSHV positive cells. (A-D) SNAIL binding region "CACCTG" were mapped on the promoter region of known binding partner, p21, SNAIL, pTEN and TGFB3 respectively. (A-D) HEK-293-BACKSHV generated recombinant virus was used to infect PBMCs and ChIP performed for SNAIL at the selected binding regions on days 2 and 5 post infection. We also included BC-3 generated virus to infect PBMCs and also subjected to binding analysis on day 2 for p21, SNAIL, pTEN and TGFB3, respectively. (E) The Promoter activity of p21 was investigated in reporter assays in the presence of sh-SNAIL and sh-Control compared to pGL3-p21 alone in BJAB cells. (F) Western blot analysis was run for the confirmation of SNAIL knockdown compared to vector control.

For a more detailed understanding of the targets of SNAIL in KSHV induced tumors, we investigated the transcription activity of SNAIL on selected promoter regulatory elements. Further, we identified a number of transcriptional target genes of SNAIL along with SNAIL binding sequences in the upstream regulatory regions of genes including p21, pTEN, TGFβ3 and the upstream region of SNAIL itself (Fig 9A–9D). Earlier studies identified the "CACCTG" signature sequence as the DNA binding sequence of SNAIL [52], and also reported the importance of SNAIL as a transcription factor for regulation of cellular genes p21, pTEN and TGFB3 [53,54,55]. Therefore, we analyzed the upstream region of these genes to identify the binding sequence of SNAIL in KSHV positive cells. We designed multiple primers within the promoter regions for these genes based on their binding residues (Table 1). This study was investigated using two physiologically relevant approaches. First, we infected PBMCs with BAC-KSHV purified virus and performed ChIP using SNAIL antibody on day 2 and 5 post-infection (Fig 9A–9D). In our second approach, we performed ChIP for SNAIL in BC-3 cells (Fig 9A–9D). Surprisingly, anti-SNAIL antibody was enriched for DNA within p21 regulatory binding regions 3 and 4 along with SNAIL regulating binding regions 4 and 5, respectively (Fig 9A and 9B). These regions were found to be enriched for SNAIL binding sites in KSHV infected cells compared to uninfected PBMCs. These results suggest that SNAIL can regulate p21 expression in infected B-cells and can contribute to KSHV induced B-cell oncogenesis. Interestingly, pTEN and TGFB3 did not show any detectable differences in KSHV induced B-cell lymphoma when compared to KSHV infected PBMCs (Fig 9C and 9D). Additionally, we probed the promoter activity of p21 in BJAB cells (Fig 9E). As expected SNAIL knockdown led to upregulation of the p21 promoter activity compared to control vector (Fig 9E). This result clearly emphasized the effect of SNAIL binding at the promoter region of p21 important for its regulation. Knockdown of SNAIL was confirmed through Western blot (Fig 9F). GAPDH was used as a protein loading control (Fig 9F).

**Table 1:** Primer sequence for ChIP analysis.

Gene	Strand	Sequence	Region
P21	FP	CTGAGGGGAGGCTCATACTG	(-3997–3978)
1.	RP	AGAGAGGCATCCTCCAGACA	(-3807-3788)
2.	FP	GAGGATGCCTCTCTTCAAAC	(-3801–3782)
	RP	CTTTGTTGGTCATCACACCT	(-3642–3623)
3.	FP	ACGGAGTCTCACTCTGTCAC	(-3342–3323)
	RP	GCACCTGTAGTCCCAGGTAT	(-3235–3216)
4.	FP	GTGGGGAGTATTCAGGAGAC	(-957–938)
	RP	CTCCAGGGAAACAGAAGAAT	(-825–806)
5.	FP	AAACGGGACTGAAAAATCAT	(-690–671)
	RP	GCTGAAAAGATCAGGAGGAT	(-503–484)
**TGFB-3**	FP	ATGGTCCTTCTGCTTCTTCT	(-452–433)
	RP	GGATTCTTGTCCATGTGTCT	(-246–227)
**PTEN 1**	FP	ATCTAGGGGTAGAGGCAAGG	(-1467–1448)
	RP	GTGGAGGACTGATGATGAAA	(-1308–1290)
**SNAIL**	FP	CTGAGGGGAGGCTCATACTG	(-3997–3978)
1.	RP	AGAGAGGCATCCTCCAGACA	(-3807-3788)
2.	FP	GAGGATGCCTCTCTTCAAAC	(-3801–3782)
	RP	CTTTGTTGGTCATCACACCT	(-3642–3623)
3.	FP	ACGGAGTCTCACTCTGTCAC	(-3342–3323)
	RP	GCACCTGTAGTCCCAGGTAT	(-3235–3216)
4.	FP	GTGGGGAGTATTCAGGAGAC	(-957–938)
	RP	CTCCAGGGAAACAGAAGAAT	(-825–806)
5.	FP	AAACGGGACTGAAAAATCAT	(-690–671)
	RP	GCTGAAAAGATCAGGAGGAT	(-503–484)

### SNAIL is involved in NF-kB mediated signaling in KSHV positive cells

**Fig 10:**
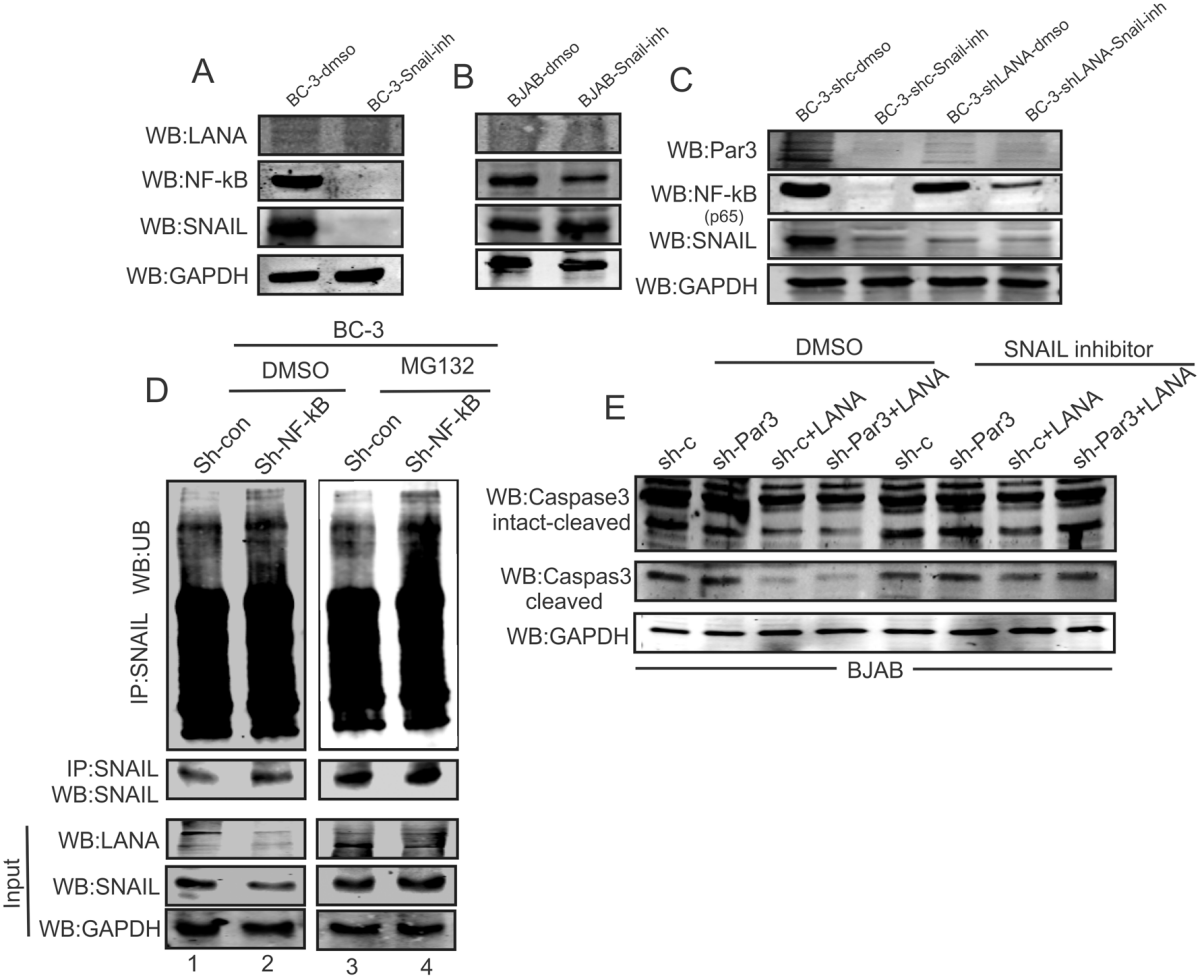
NF-kB is regulated by SNAIL in KSHV positive cells. (A) BC-3 and (B) BJAB cells were treated with DMSO and SNAIL inhibitor for 48 hours and assessed through Western blots for LANA, NF-kB, SNAIL and endogenous GAPDH. (C) BC-3 shControl and BC-3-ShLANA were treated with DMSO and SNAIL inhibitor for 48 hour and blotted for Par3, NF-kB (p65), SNAIL and endogenous control GAPDH. (D) Ubiquitination assays for SNAIL were performed in BC-3sh-control and sh-NF-kB cells treated with DMSO and MG132. (E) sh-Control and sh-Par3 in presence/absence of LANA were measured in the BJAB cell background. Left panel was observed in the presence of DMSO and the right panel was treated with SNAIL inhibitor at the same time. Caspase3 and GAPDH was observed in both panels.

SNAIL can regulate itself and deregulates p21 in KSHV positive cells. It was important to explore other downstream targets which may be important for oncogenesis. Additionally, previous studies demonstrated the importance of NF-kB in KSHV-induced tumors [56]. Additional studies showed that p21 down-regulation was found to be critical for NF-kB-mediated oncogenesis [57]. This prompted us to follow-up on our current data above to more closely understand the link between p21 and NF-kB in KSHV positive cells. Interestingly, in other studies we also observed that small molecules inhibitors targeted EBV and KSHV lymphoma cells through the NF-kB pathway [58]. Here we analyzed BC-3 cells in the presence of a SNAIL inhibitor, as a KSHV positive cell line compared to the KSHV negative cell line BJAB (Fig 10A and 10B). Interestingly, we observed that in the presence of a SNAIL inhibitor the levels of NF-kB (p65 and p50) in BC-3 cells was significantly diminished. However, this reduction was significantly less in KSHV negative BJAB cells (Fig 10A and 10B). Similarly, NF-kB (p65) levels were substantially down in sh-LANA, compared to vector control in BC-3 cell background (Fig 10C). Earlier, several studies suggested that NF-kB can regulate SNAIL at the transcriptional and post-translational levels [59]. To investigate if this holds up in KSHV positive cells we utilized the proteosome inhibitor MG132 and DMSO control in BC-3 sh-Control and sh-NF-kB cells (Fig 10D). Interestingly, our results revealed that in KSHV positive cells NF-kB knockdown led to increased ubiquitination of SNAIL, which may explain the post-translational regulation of SNAIL by NF-kB in a KSHV positive background. A similar pattern was observed in sh-Control when compared to the NF-kB knockdown cells in DMSO treated cells, suggesting a role for NF-kB in SNAIL degradation (Fig 10D, compare lane 1 and 2 with 3 and 4). These results strongly demonstrated the importance of NF-kB in SNAIL regulation in KSHV-infected cells. We also showed that LANA knockdown reduced the impact of the SNAIL inhibitor when compared to vector control which strongly suggests a critical role for LANA in SNAIL-mediated gene regulation during KSHV infection (Fig 10A–10D). Furthermore, we also monitored Caspase3 cleavage in sh-Control and sh-Par3 in the presence and absence of LANA, and a SNAIL inhibitor (Fig 10E). Surprisingly, the presence of LANA reduced Caspase3 cleavage in BJAB cells (Fig 10E). However, the SNAIL inhibitor induced Caspase 3 cleavage in the presence as well as the absence of LANA (Fig 10E). These results suggested that NF-kB is positively regulated by SNAIL whereas Caspase 3 is negatively modulated by SNAIL in KSHV positive cells. We have also seen the minimal variation of v-Flip and v-Cyclin in LANA knockdown BC-3 and JSC1 cells (S4A and S4B Fig).

### SNAIL inhibition induced E-Cadherin expression in KSHV positive cells

**Fig 11:**
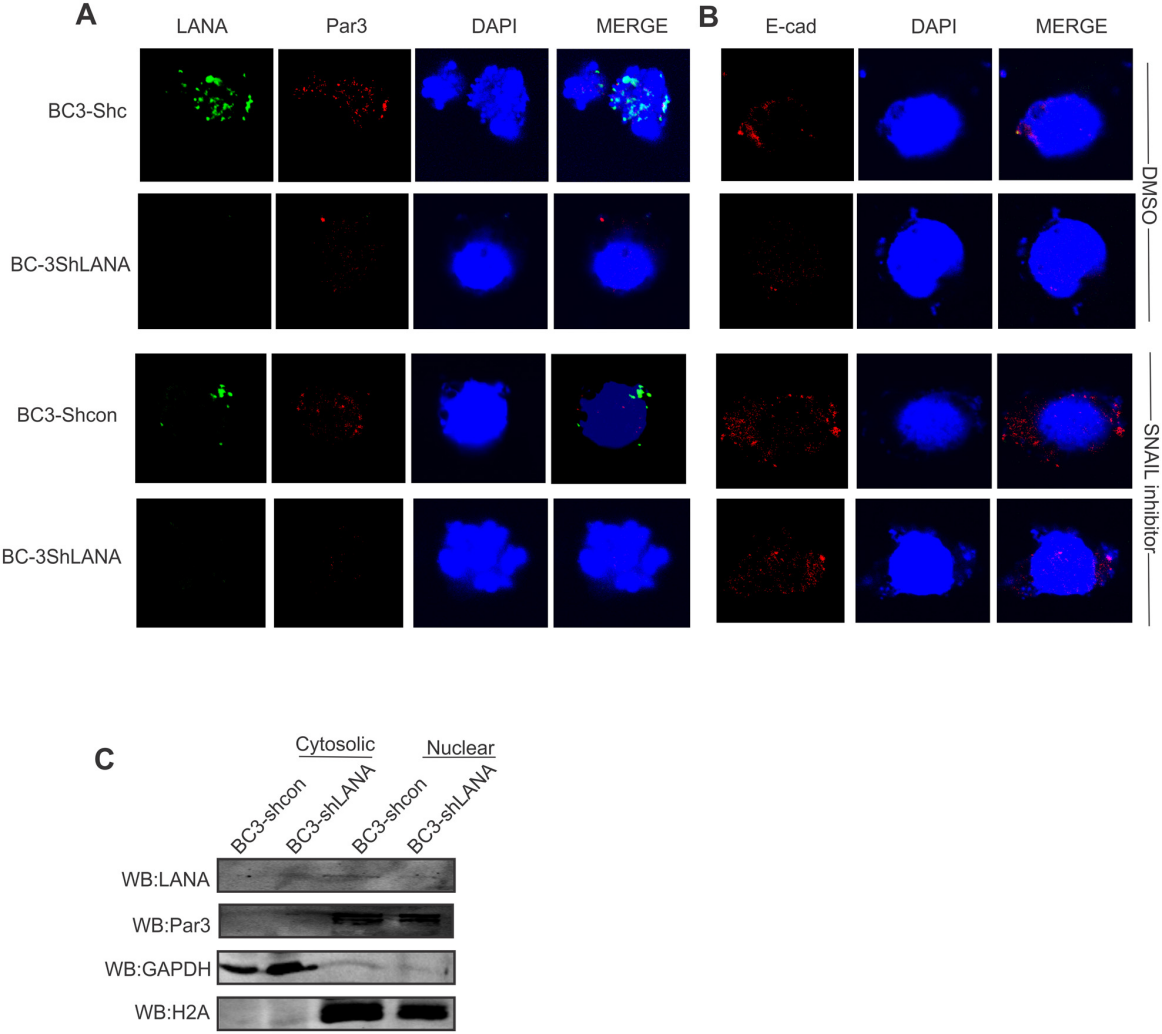
E-Cadherin levels are induced by a SNAIL inhibitor in KSHV positive cells. (A, B) BC-3 shControl and BC-3 sh-LANA cells were treated with DMSO and SNAIL inhibitor and immunostained with (A) LANA, Par3, and nuclear stain DAPI (B), E-Cadherin and nuclear stain with DAPI. (C) Cell fractionation assays were carried out in BC-3Shcontrol and BC-3shLANA cells. GAPDH was used as a control for cytosolic fraction and H2A was used as a control for nuclear fraction.

Previous results demonstrated that KSHV infection led to up-regulation of SNAIL and down-regulation of E-Cadherin [29]. Moreover, SNAIL inhibition in KSHV positive BC-3 led to E-Cadherin upregulation providing a direct link between SNAIL and E-Cadherin regulation in KSHV positive cells (Fig 11B). Earlier, we showed that knockdown of SNAIL led to downregulation of Par3 and upregulation of E-cadherin. Here we showed that by using a SNAIL inhibitor *in vitro* the expression of Par3 in BC-3 cells was dramatically reduced as seen by immunofluorescence (Fig 11A). Additionally, E-cadherin signals by immunofluorescence was increased supporting our earlier studies above (Fig 11B). Using cell fractionation assays, Par3 was selectively present in the nuclear compartment with LANA. However LANA knockdown in BC-3 cells lads to transfer of some of the Par3 signals in to the cytoplasmic fraction (Fig 11C). Here GAPDH was used as a loading control for cytoplasmic fractions and H2A was used for the nuclear fraction (Fig 11C). LANA was shown as a positive control for KSHV positive BC-3 cells.

## Discussion

Kaposi Sarcoma (KS) is an endothelial tumor typically infected with HHV-8 [60]. Immune-suppression is tightly associated with KS development and remains the second most frequent tumor seen in acquired immune deficiency syndrome patients [61]. Earlier, it was suggested that Par3 functions as a potential “gatekeeper” to sustain the phosphoinositide concentration gradient in polarized cells [62]. Importantly, polarization is a crucial step towards progression of epithelial cells to mesenchymal cells [24]. We evaluated several DNA damage markers known to be critical for viral induced oncogenesis. Surprisingly, Par3 which was shown to be important for cell polarization, was significantly upregulated in the presence of KSHV. We also found significant changes in Par3 levels with KSHV infection of primary B-cells at the transcript and protein levels. This expression increased as the infection progressed. Earlier, we established that KSHV lytic genes are essential for virus infection and increased from day 2 to 6 [63]. Further, in KSHV infected B-cell lines (BC-3 and BCBL1) and with BAC-KSHV infected cells a similar trend was observed compared to control. Interestingly, knockdown of LANA directly inhibited the basal expression levels of Par3 in KSHV positive cells.

A prominent association was demonstrated between the 373–653aa residues of the LANA-N-terminal and Par3 when compared to C-terminal (611–1266aa) which had a much lower capacity to bind LANA. Furthermore, we showed a high degree of co-localization between LANA and Par3 in B-cells. These results suggest a possible functional regulation of Par3 by LANA in KSHV-infected cells.

Earlier studies from our lab and others demonstrated that LANA can regulate host proteins through protein stabilization or degradation during KSHV infection [64,65,66,67]. To examine Par3 modulation in the presence of LANA we performed stabilization and dose-dependent assays. We observed that Par3 degradation was significantly delayed in the presence of LANA when compared to controls. Earlier studies showed that many cellular proteins are stabilized by LANA through a similar mechanism [68]. Furthermore, localization of proteins are directly linked to their functional roles and their pattern of expression are typically linked to their functional relevance [68]. Interestingly, Par3 is a large protein (180 kDa), which needs an active process to be transported to the nucleus for signaling [69]. Our results suggested that LANA induced Par3 translocation to the nucleus in KSHV-infected cells. We found a localization pattern in endogenously infected B-cells, where Par3 signals were peripheral in KSHV negative cells, compared to a predominantly nuclear signal in KSHV positive cell lines. This result was validated in KSHV infected PBMCs. Furthermore, Par3 knockdown led to arrest of cell proliferation and enhanced apoptosis. These results are similar to that observed for other well known oncoproteins strongly supporting a role for Par3 as an oncoprotein which contributes to KSHV-induced cell transformation [48].

Pathogenic viruses have evolved mechanisms to disrupt adherence junctions, important for maintenance of the integrity of the endothelium [70]. This disruption of junctional barriers ensures that the viruses can gain entry through the open receptors in the basolateral membranes and so traffics to other tissues through the lymphatic and vascular networks [71]. Qian et al, demonstrated that KSHV infection induces vascular permeability by down-regulating VE-cadherin expression to allow virus entry into cells [11]. Moreover, KSHV-mediated aberrant vascular permeability likely stimulates virus replication, inflammation and angiogenesis, which enhances pathogenesis of KSHV-induced malignancies. Furthermore, loss of E-cadherin increases expression of MMPs, one of the hallmark for EMT progression [72]. However, how these EMT markers are modulated and functionally involved in B-cell associated lymphomas was not extensively explored. Recently, two separate studies from Lemma et al., and Tillo et al demonstrated the importance of EMT markers in B-cell lymphoma and mantle cell lymphoma respectively [21,22].

Therefore, evaluation of these EMT markers as crucial indicators of ECM stability and strength is an important component of the many cellular processes which contribute to KSHV-induced oncogenesis. ECM signaling modulated by LANA expressed from BAC-KSHV after infection showed that expression of E-cadherin was down-regulated in BAC-KSHV infected cells, whereas expression of SNAIL was enhanced. These results suggested a direct association between KSHV infection and EMTs. Additionally, we screened a series of EMT markers in Par3 knockdown B-cells. Surprisingly, the majority of epithelial markers were enhanced whereas mesenchymal markers were down-regulated. Moreover, SNAIL was dramatically modulated in Par3 knockdown, and virus infected B-cells. These results emphasizes the importance of SNAIL in KSHV-induced B-cell oncogenesis. We further corroborated the importance of SNAIL in KSHV infection in primary B cells by demonstrating that SNAIL is directly regulated by Par3 and LANA and can also deregulate E-cadherin for establishment of KSHV latent infection. During EMT, MMPs-mediated E-cadherin disruption is a determining process where E-cadherin is essential for transmitting signals to epithelial cells through catenin, SNAIL and Slug [73]. Furthermore, in gastric cancer SNAIL was shown to affect the invasiveness and migratory ability of cancer cells during metastasis [30].

SNAIL is a zinc-finger transcriptional repressor, which directly represses E-cadherin through regulation of the E-cadherin promoter [74]. It is also recognized as an important regulator in various cancer including ovarian, non-small cell lung, gastric, hepatocellular, and urothelial [75,76]. Moreover, SNAIL has been shown to induce VEGF and MMPs, well-established tumor invasion and metastasis markers [30]. In this study we found that SNAIL knockdown and its specific inhibitor diminished the expression of LANA suggesting a level of regulation also at the viral genome, as well as Par3, while E-cadherin was moderately enhanced in B-cells. We also demonstrated the importance of SNAIL as a transcription factor where it binds its own sequence within its promoter, as well as the promoter region of p21. Earlier, p21 down-regulation was shown to be important in Herpesvirus infection [31]. Although, NF-kB a well-known signaling molecule is enhanced in KSHV-induction, it is also reduced significantly in the presence of a SNAIL inhibitor in KSHV positive cells compared to KSHV or LANA negative cells, robustly supporting our hypothesis.

In this study we showed that LANA regulates Par3 activity and EMT markers important for metastasis of KSHV-infected cells. This is similar to other viral oncogenes like E6 of human papilloma virus (HPV) which also targets the cell-polarity proteins Dlg and Scrib for proteolytic degradation [77]. However, a study by McCaffrey et al demonstrated that in breast cancer Par3 was shown to be tumor suppressor [78]. In KSHV infection, LANA can therefore functions as a master protein which regulates both transcription and cell growth during metastasis of KSHV-infected cancer cells and can also modulate gene expression during KSHV latent infection [79]. Further, a large number of cellular and viral promoters are regulated by LANA through its repressing or activating activities [45,48,64].

**Fig 12:**
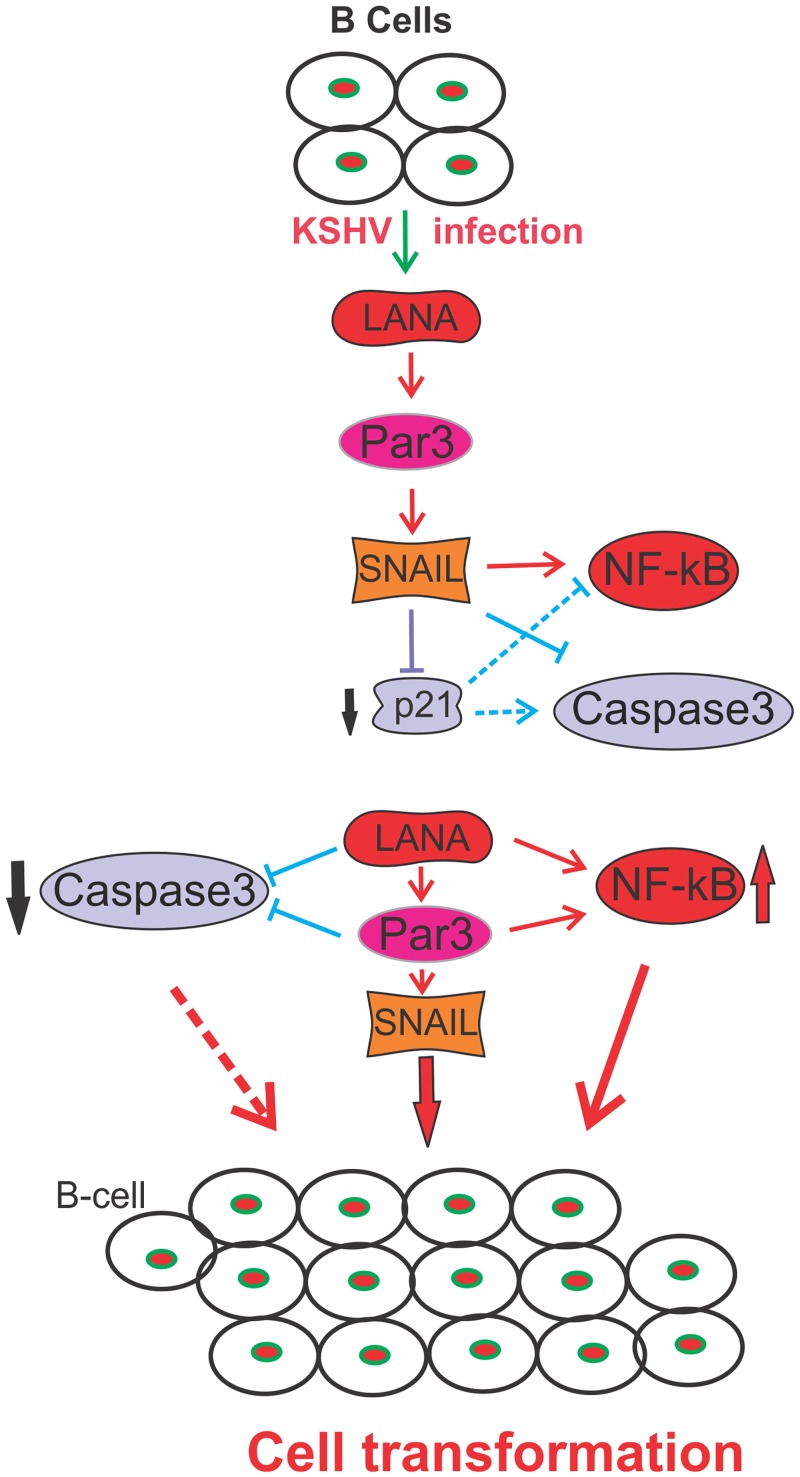
Schematic represents a putative model illustrating the contribution of Par3 and SNAIL to KSHV-associated cancers. KSHV infected endothelial or B-cells expresses LANA important for establishment of latent infection. LANA up-regulates Par3 and SNAIL which leads to epithelial to mesenchymal transition through down-regulation of E-cadherin and enhanced expression of MMP9 in B-cells. This model suggests that KSHV-infection can regulate the EMTs important for progression of infected cells to an oncogenic and invasive phenotype.

Overall, KSHV-infection results in expression of LANA which leads to induction of Par3 and SNAIL expression in B-cells. This enhances MMP9 expression and reduces E-cadherin levels (Fig 12). Further, SNAIL binds to p21 and up-regulates NF-κB activities through induction of Caspase3 cleavage and suggests a potential mechanism through which LANA can regulate the EMT markers important for transition and invasion of KSHV-infected B-cells through the extracellular matrix (Fig 12).

## Materials and Methods

### Ethics statement

PBMC were obtained from University of Pennsylvania Human Immunology Core (HIC) and donated by the healthy donors. This study was approved by University of Pennsylvania Human Immunology Core which maintains IRB protocol. In this IRB approved protocol declarations of Helsinki protocols were followed and each donor gave written, informed consent [48]. The mice studies were conducted under the project entitled "therapeutic small molecule inhibitors of EBV and KSHV". All six-week-old male NOD/SCID mice were purchased from (Jackson Labs, Bar harbor, ME, USA). The office of Laboratory Animal Welfare (OLAW) guidelines provided by National Institute of Health (NIH) USA, are strictly followed by the University of Pennsylvania Institutional Animal Care and Use Committee (IACUC). Our protocol number for this study was 804617 and we have adhered with all OLAW guidelines.

### Plasmids, cells and antibodies

Par3 was cloned from human PBMCs. Primers with specific restriction enzyme sites for cloning in pA3HA vector was used for amplification of specific product for cloning. The pA3F-LANA expresses FLAG-tagged LANA. pA3M-LANA, pA3M-N-LANA and pA3M-GFP-C-LANA express Myc-tagged, full length LANA, N-terminal of LANA (1–340 AA) and C-terminal of LANA (930–1162) with GFP-tag respectively, and have been described previously [64]. For expression of GST fusion protein, cDNA from PBMCs was amplified using primers specific for Par3 amplicon and cloned in pGEX2T vector. An sh-SNAIL and control construct was provided by Dr. Gerhard Christofori (University of Basel, Basel, Switzerland). Sequences of all the constructs were verified by DNA sequencing (DNA sequencing facility, University of Pennsylvania). HEK-293 (human embryonic kidney cell line) was obtained from Jon Aster (Brigham and Woman's Hospital, Boston, MA). KSHV-negative Burkitt’s lymphoma cells Ramos and BJAB were kindly provided by Elliot Kieff (Harvard Medical School, Boston, MA). BC3 and BCBL1 are KSHV-positive, lymphoma-derived cell lines obtained from the American Type Culture Collection (ATCC). JSC-1 cells is a latently infected KSHV B-lymphoma cell line, kindly provided by Richard F. Ambinder (John Hopkins, Baltimore, Maryland). Wild-type KSHV BACmid was a kind of gift from Shou-Jiang Gao (University of Texas, San Antonio, TX). KSHV-negative cell line Ramos, BJAB, and the KSHV-positive cell lines BC-3, BCBL1 and JSC-1 were cultured in RPMI 1640 medium with 10% bovine growth serum (BGS) with additional supplements as described previously [64]. Human embryonic kidney 293 (HEK-293) and HEK-293-BACKSHV cell lines were cultured in Dulbecco's modified Eagle's medium (DMEM) supplemented with 5% BGS, penicillin-streptomycin (5U/ml and 5μg/ml, respectively), and 2 mM L-glutamine. Primary antibodies for SNAIL (H-130), MMP9 (SC-1609), E-cadherin (H-108), NF-kBp65 (SC-109) NF-kBp50 (SC-114), and Caspase3 (SC-7272) were purchased from Santa Cruz Inc. (Santa Cruz, CA, USA). Anti-Rabbit polyclonal Par3 (07–330) antibodies were purchased from Millipore (Massachusetts, USA). Hybridoma culture supernatants were used as sources of anti-Myc (9E10) and anti-LANA (LANA1). Mouse anti-FLAG monoclonal antibody (M2) was purchased from Sigma-Aldrich Corp., (St. Louis, MO).

### KSHV virus production and transduction of PBMCs

Induction of KSHV from HEK-293-BACKSHV procedure were followed as described earlier [48]. The KSHV virus was collected by centrifugation at 23,500 rpm for 1.5 to 2 hr at 4°C by ultracentrifugation. KSHV-infection was performed by incubation of primary cells (PBMCs) with virus using 4 μg/ml polybrene in RPMI 1640 medium (10% BGS) and 5% CO_2_ at 37°C for 3–4 hr. KSHV-infection was assessed by evaluating the expression of GFP using fluorescence microscopy.

### Real-time PCR

Total RNA was isolated from experimental cells using TRIzol reagent Invitrogen Inc. (Carlsbad, CA) as per manufacturer's instructions. All procedures were followed as described earlier [39]. Step-one Real-time PCR Cycler was used with the default program settings. A melt curve analysis was also performed to ensure the specificity of amplified products. Relative quantitation was carried out by the threshold cycle method. All reactions were set up in triplicates. Primers used in these studies are listed in Table 1.

### GST-pull down assays

For expression of GST and GST-fusion proteins, bacterial cultures were induced with 1 mM IPTG at log phase (OD600 = 0.6) and incubated with shaking at 37°C for 4 hr. *E*. *coli* BL21-DE3 cells were used for all transformation in this experiment. Bacterial lysis and purification of proteins with Glutathione-Sepharose beads were carried out as described earlier [39]. In brief, the cultures were pelleted at 3000 RPM for 10 minute and the pellets washed with STE buffer and resuspended in NETN buffer (100 mM NaCl, 20 mM Tris-Cl (pH 8.0), 0.5 mM EDTA 0.5% (v/v), Nonidet P-40). Dithiothreitol (DTT) and 10% Sarkosyl in STE buffer (100mM NaCl, 10 mM Tris, 1 mM EDTA) were mixed into the solution, and the sample was sonicated to solubilized the proteins. The lysates were collected by centrifugation and the supernatant transferred and followed by addition of Triton X-100 in STE and Glutathione-Sepharose with rotation. GST-fusion proteins, bound to Glutathione-Sepharose beads were incubated with cell lysates to allow binding of GST-fusion protein with the target protein. After stringent washes the beads were boiled in SDS-sample loading buffer to allow denaturation and dissociation of bound proteins. Samples were centrifuged and the supernatant were subjected to SDS-PAGE fractionation followed by western blotting.

### Co-immunoprecipitation (Co-IP) and western blot (WB) assays and analysis

25 million cells were used for immunoprecipitation (IP) of exogenously expressed proteins using specific mono or polyclonal antibodies as described earlier [39]. In brief, the cell lysates from experimental groups were incubated similar to controls with species specific IgG and beads as well as gene specific primary antibodies and beads. A mixture of Protein A/G-Sepharose beads (1:1) was used in these assays. For removal of non-specific binding, stringent washes were carried out with RIPA buffer. Further the conjugated beads were boiled in 4x SDS-sample loading buffer and subjected to SDS-PAGE gel for western-blot experiments. Western blot protocols and quantitation of bands were performed as described earlier [39]. The membrane was blocked with 5% skim milk in phosphate-buffered saline (PBS) for 1 hour, followed by incubation with the appropriate dilution of primary antibodies (in PBS) for 2 hour at RT. IR dye-tagged secondary antibodies were used to detect the binding of primary antibodies, and the membrane was scanned using an Odyssey imager (LiCor Inc., Lincoln, NE). Quantitation of the protein bands was carried out using Odyssey scanning software.

### Immunoflorescence (IF) assays and analysis

IF experiments were essentially carried out as described earlier [45]. In brief, experimental cells were washed two times with ice cold PBS. Four percent paraformaldehyde containing 0.1% TritonX-100 were used to fix and permeabilize the cells for next steps. Again cells were washed with ice cold PBS and blocked using freshly made 5% skim milk for 1 hr. For LANA and Par3 co-localization experiments, we used Ramos, HEK-293, BJAB, BCBL1, BC-3, JSC-1, BC-3-shControl, BC-3-shLANA and 293BAC-KSHV cells. HEK-293 and 293BAC-KSHV cells were used to evaluate the localization pattern for Par3, E-cadherin, SNAIL, MMP9 and LANA. All primary antibodies as mentioned earlier were used for specific staining in cells. Evaluation of nuclear staining with different sets of proteins were carried out with DAPI (4,6-diamidino-2-phenylindole). After being washed with PBS, coverslips were mounted on glass slides using mounting medium. An Olympus Fluoview 300 confocal microscope was used in all experiments.

### RNA interference

pGIPZ vector was used for gene specific shRNA cloning. This vector was purchased from Open Biosystems, Inc. (Huntsville, AL). Oligos for shPar3 clone was designed against a unique region of Par3: 5’ CAACAAGAAGACGCGAATC 3’ and LANA: 5' GCTAGGCCACAACACATCT 3'. A shRNA sequence with no significant homology with other human mRNA was used as a sh-control and was described earlier [80]. To monitor the knockdown effect of SNAIL, we used shSNAIL and control shRNA as described previously [81].

### Stability assays

HEK-293, BJAB control and BJAB stably expression with LANA cells were used for this assay. In HEK-293, 36 hours post-transfection, cells were exposed to 40 μg/ml cyclohexamide (CalBiochem, Gibbstown, NJ) at given time points. For BJAB cells, 25 million cells were incubated with 100μg/ml cyclohexamide in normal serum medium. 20μM/ml MG132 concentration was used for treating the BJAB control and BJAB with stable expression for LANA. Subsequently, proteins were prepared at given time points and analyzed by immunoblotting with suitable antibodies. Odyssey Imager (LiCor Inc., Lincoln, NE) was used to measure band intensities.

### Immunohistochemistry (IHC) assays

All procedures for IHC were followed as described earlier [48]. Paraffin-embedded tissues were sectioned at the New Bolton Center, Animal Pathology Service, University of Pennsylvania School of Veterinary Medicine. Slides were deparafinized in Xylene. Treatment with H_2_O_2_ was followed by antigen retrieval in sodium citrate buffer (pH 6.0), and samples were blocked with 10% normal rabbit/goat serum prior to incubation with primary antibodies O/N at 4°C. Further procedures were followed as described earlier [48]. In brief, evaluation of nuclear staining was carried out with DAPI (4,6-diamidino-2-phenylindole) in mix with secondary antibodies for 1 hour at RT. After being washed with PBS, coverslips were mounted on glass slides using mounting medium. An Olympus Fluoview 300 confocal microscope was used in all experiments.

### Colony formation assays

HEK-293 cells with pGIPz-shControl, pGIPz-shPar3 and vector control or LANA plasmids were transfected and placed in petri dishes and grown in culture medium containing puromycin and G418 antibiotic selection (2μg/ml). All procedures were followed as reported earlier [39]. Culture medium with antibiotics were replaced every alternate day. After two weeks in culture, cells were washed with PBS and fixed in 4% PFA for 30 minutes at room temperature in the dark. Cells were stained with 0.1% crystal violet (Sigma-Aldrich Corp., St. Louis, MO). Plates were washed to remove non-specific color and photographed using an Odyssey scanner (LiCor Inc., Lincoln, NE). The number of colonies are representated as a percentage using Image J software (National Institutes of Health, Bethesda, MD).

### Colony growth assays

HEK-293 cells with pGIPz-shControl, pGIPz-shPar3 and vector control or LANA plasmids were transfected and placed in petri dishes and grown in culture medium. BC-3 and BCBL1 cells with pGIPz-shControl and pGIPz-shPar3 were transfected and placed in T25 flask and grown in culture medium in 6 well plates. 48 hour post transfection 50,000 cells were plated for day1, day2 and day 3 analysis. According to time points, cells were washed with PBS and fixed in 4% PFA for 30 minutes at room temperature in the dark. Cells were stained with 0.1% crystal violet (Sigma-Aldrich Corp., St. Louis, MO). Plates were washed to remove non-specific color and photographed using an Odyssey scanner (LiCor Inc., Lincoln, NE). B-cells were subjected to scan directly after completion of time points. The number of colonies are representated as a percentage using Image J software (National Institutes of Health, Bethesda, MD).

### Cell cycle and apoptosis assays

Stable cell line for sh-control and sh-Par3 in HEK-293 cells were used in this study. Before harvesting an equal number of cells were kept with 0.1% serum with culture medium for 12 hr. Further cells were harvested by trypsinization, washed three times with PBS and fixed in 1:1 ratio of methanol: acetone for 12 hr at 4°C. Cells were incubated with 200 μg/ml of RNase A and kept in the -20°C freezer for 3 hr. Cells were then stained with propidium iodide (PI) 40 μg/ml (Sigma, St Louis, MO) and dissolved in PBS containing at least 1 hr at 4°C in the dark. In etoposide treated experiment, cells were incubated with etoposide (20 μM/mL) for 12 hr and a similar protocol followed as described for serum starved cells. Cells in different cell cycle phases with appropriate controls were differentiated through FACS Calibur (BD Biosciences, San Joe, CA) and the results were assessed by the FlowJo software (Tree star, Ashland, OR).

### Proliferation assays

HEK-293 cells were transfected using Ca_3_(PO4)_2_ method as described [82]. Twenty four hour post transfection, cells were selected using puromycin (2μg/ml) added to the culture media. Approximately 0.1 million cells from both sets of samples were plated into each well of the 6-well plates and cultured for 6 days. All procedures were followed as described earlier [80]. BC-3 and BCBL1 cells with pGIPz-shControl and pGIPz-shPar3 were transfected and placed in T25 flask and grown in culture medium. 48 hour post transfection cells were counted with Trypan blue dye on days dependent manner.

### Luciferase reporter assays

Reporter assays were essentially performed as described previously [49]. In brief, plasmid mixes were prepared according to experiments, and control plasmids were added as needed to normalize the amount of total DNA. Following transient transfection, cells were incubated for 48 hours and lysed in reporter lysis buffer (Promega Inc., Madison, WI). Aliquots of the lysates were transferred to 96-well plates, an luciferase activity monitored as described earlier [49].

### Ubiquitination assays

Thirty million BC-3-shControl and BC-3-shLANA cells were immunoprecipitated with 1 μg of anti-Par3 antibody and Western blotted with anti-ubiquitination, Par3, LANA, and GAPDH antibodies. Cells were incubated for 12 h with 50 μM MG132 (Santa Cruz, Inc., Dallas, Texas). All procedures were followed as described earlier [49]. Thirty million of BC-3-shControl and Bc-3-shLANA were immunoprecipitated with 1 μg of anti-SNAIL antibody and Western blotted with anti-ubiquitination, SNAIL, LANA, and GAPDH antibodies.

### *In vivo*: Analysis of Par3 and SNAIL expression

Six-week-old male NOD/SCID mice (Jackson Labs, Bar harbor, ME, USA) were acclimatized for one week and divided into two groups (5 mice per group). Representative pictures of results are shown in Fig 7. Five mice were injected with BC-3 (KSHV positive cells) and other five mice were injected with BJAB (KSHV negative cells) cells intraperitoneally. Prior to injection, each mouse was anesthetized by intraperitoneal administration of ketamine (80mg/kg) and xylazine (5mg/kg) mixture. Five weeks later, mice from all groups were euthanized when the tumor size grew to more than 15 mm in size. All mice were necropsied to determine gross metastases. Tumors were preserved in 10% formalin for histopathology, mRNA and protein extraction and antigen detection.

### Chromatin immunoprecipitation (ChIP)

Chromatin immunoprecipitation (ChIP) was carried out as has been described earlier [49]. Briefly, after the cells were fixed with formaldehyde, cells were washed with cell lysis buffer. Followed by nuclei isolation and sonication in the nuclear lysis buffer to an average DNA length of 300–700 bp, as confirmed by agarose gel electrophoresis. Samples were precleared with salmon sperm DNA-protein A-Sepharose slurry for 1 hr at 4°C with rotation. Twenty percent of the total supernatant was saved for input control, and the remaining 80% was divided into two fractions and incubated with (i) control antibody (Sigma, Inc., St. Louis, MO) or (ii) Poly-clonal anti-Rabbit Par3 antibody. The precipitated immune complex was washed for stringency, reverse cross-linked, and purified using a phenol: chloroform extraction method.

### SNAIL inhibitor analysis

A p53-Snail binding Inhibitor, GN25 from CalBiochem (Merck K Darmstadt, Germany) was purchased and used in this study. SNAIL inhibition study was followed as described in an earlier study [83]. It has been demonstrated that p53 is suppressed and eliminated from cells by direct binding with oncogenic K-Ras-induced SNAIL [82]. Further they generated specific inhibitors against p53-Snail binding (GN25). This chemicals can induce p53 expression. Moreover, GN25 can selectively activate wild-type p53 in p53WT/MT cancer cells. *In vivo* xenograft test also supports the antitumor effect of GN25.

## Supporting Information

S1 Fig Par3 staining in vector control transfected Ramos cells. Ramos cells were transiently transfected with vector alone and Par3 in the absence of LANA. Par3 staining was predominantly localized at the cell periphery and inner cytoplasmic membrane.
(TIF)

S2 Fig Par3 knockdown leads to a delay in cell proliferation and induction of apoptosis. (A) Colony formation assays were carried out under puromycin antibiotic selection using HEK-293 cell lines. Representative graphs were also plotted for every set of experiments. Quantification was based on the % of colonies as 100 in control plates compared to the Par3 knockdown. (B) Cell proliferation assays monitored using cell counting from day 1 to 6 in HEK-293 cells. Graphs were drawn as number of cells per thousand for sh-control and sh-Par3 -BAC-KSHV cells. (C) Real-time PCR was performed to determine the efficiency of Par3 knockdown at the transcript levels. (D) HEK-293 cells were used to study the resistance from apoptosis after serum starvation. sh-Par3 and sh-control plasmids were transfected for 24 hr followed by serum starvation for 24 hr. Propidium iodide (PI) staining was performed with flow cytometry. Graphs represents the phases of the cell cycle. G0 phase of cells were determined as cell death population. (E) HEK-293 cells were transfected with sh-control and sh-Par3 for 24 hr followed by 12 hr etoposide treatment. PI staining was examined by flow cytometry. (F) A representative graph in fold change explaining the cell population in G0 phase either in serum starvation or etoposide treatment compared to control for HEK293-shControl and HEK293-shPar3.
(TIF)

S3 Fig Expression of LANA and Par3 in B-cells. (A) LANA and Par3 expression were checked for LANA and Par3 in exogenous expressed transfected cells for LANA and Par3 sh construct. GAPDH was used as endogenous control. (B and C). Par3 expression was assessed in BC-3 and BCBL1 cells transfected with Par3sh and control. GAPDH was used as endogenous control.
(TIF)

S4 Fig Expression of v-Cyclin and v-Flip in LANA knockdown BC-3 and JSC-1 cells. (A-B) BC-3 and JSC-1 LANA knockdown compared to vector control cells were evaluated for LANA, v-Cyclin and v-Flip transcript expression. qRT-PCR was performed with cDNA samples.
(TIF)

## References

[1] Chang Y, Cesarman E, Pessin MS, Lee F, Culpepper J, et al (1994) Identification of herpesvirus-like DNA sequences in AIDS-associated Kaposi's sarcoma. Science 266: 1865–1869. 7997879 10.1126/science.7997879

[2] McAllister SC, Moses AV (2007) Endothelial cell- and lymphocyte-based in vitro systems for understanding KSHV biology. Curr Top Microbiol Immunol 312: 211–244. 17089799 10.1007/978-3-540-34344-8_8

[3] Nador RG, Cesarman E, Chadburn A, Dawson DB, Ansari MQ, et al (1996) Primary effusion lymphoma: a distinct clinicopathologic entity associated with the Kaposi's sarcoma-associated herpes virus. Blood 88: 645–656. 8695812

[4] Chen YB, Rahemtullah A, Hochberg E (2007) Primary effusion lymphoma. Oncologist 12: 569–576. 17522245 10.1634/theoncologist.12-5-569

[5] Elgui de Oliveira D (2007) DNA viruses in human cancer: an integrated overview on fundamental mechanisms of viral carcinogenesis. Cancer Lett 247: 182–196. 16814460 10.1016/j.canlet.2006.05.010

[6] Wang HW, Trotter MW, Lagos D, Bourboulia D, Henderson S, et al (2004) Kaposi sarcoma herpesvirus-induced cellular reprogramming contributes to the lymphatic endothelial gene expression in Kaposi sarcoma. Nat Genet 36: 687–693. 15220918 10.1038/ng1384

[7] Hengge UR, Ruzicka T, Tyring SK, Stuschke M, Roggendorf M, et al (2002) Update on Kaposi's sarcoma and other HHV8 associated diseases. Part 2: pathogenesis, Castleman's disease, and pleural effusion lymphoma. Lancet Infect Dis 2: 344–352. 12144897 10.1016/s1473-3099(02)00288-8

[8] Aboulafia DM (2000) The epidemiologic, pathologic, and clinical features of AIDS-associated pulmonary Kaposi's sarcoma. Chest 117: 1128–1145. 10767252 10.1378/chest.117.4.1128

[9] Tsai YH, Wu MF, Wu YH, Chang SJ, Lin SF, et al (2009) The M type K15 protein of Kaposi's sarcoma-associated herpesvirus regulates microRNA expression via its SH2-binding motif to induce cell migration and invasion. J Virol 83: 622–632. 10.1128/JVI.00869-08 18971265 PMC2612383

[10] Wu YH, Hu TF, Chen YC, Tsai YN, Tsai YH, et al (2011) The manipulation of miRNA-gene regulatory networks by KSHV induces endothelial cell motility. Blood 118: 2896–2905. 10.1182/blood-2011-01-330589 21715310

[11] Qian LW, Greene W, Ye F, Gao SJ (2008) Kaposi's sarcoma-associated herpesvirus disrupts adherens junctions and increases endothelial permeability by inducing degradation of VE-cadherin. J Virol 82: 11902–11912. 10.1128/JVI.01042-08 18815301 PMC2583667

[12] Qian LW, Xie J, Ye F, Gao SJ (2007) Kaposi's sarcoma-associated herpesvirus infection promotes invasion of primary human umbilical vein endothelial cells by inducing matrix metalloproteinases. J Virol 81: 7001–7010. 17442715 10.1128/JVI.00016-07 PMC1933284

[13] Mellman I, Nelson WJ (2008) Coordinated protein sorting, targeting and distribution in polarized cells. Nat Rev Mol Cell Biol 9: 833–845. 10.1038/nrm2525 18946473 PMC3369829

[14] Park SM, Gaur AB, Lengyel E, Peter ME (2008) The miR-200 family determines the epithelial phenotype of cancer cells by targeting the E-cadherin repressors ZEB1 and ZEB2. Genes Dev 22: 894–907. 10.1101/gad.1640608 18381893 PMC2279201

[15] Brown RL, Reinke LM, Damerow MS, Perez D, Chodosh LA, et al (2011) CD44 splice isoform switching in human and mouse epithelium is essential for epithelial-mesenchymal transition and breast cancer progression. J Clin Invest 121: 1064–1074. 10.1172/JCI44540 21393860 PMC3049398

[16] Ikenouchi J, Matsuda M, Furuse M, Tsukita S (2003) Regulation of tight junctions during the epithelium-mesenchyme transition: direct repression of the gene expression of claudins/occludin by Snail. J Cell Sci 116: 1959–1967. 12668723 10.1242/jcs.00389

[17] Slawson C, Hart GW (2011) O-GlcNAc signalling: implications for cancer cell biology. Nat Rev Cancer 11: 678–684. 10.1038/nrc3114 21850036 PMC3291174

[18] Thiery JP, Acloque H, Huang RY, Nieto MA (2009) Epithelial-mesenchymal transitions in development and disease. Cell 139: 871–890. 10.1016/j.cell.2009.11.007 19945376

[19] Tanos B, Rodriguez-Boulan E (2008) The epithelial polarity program: machineries involved and their hijacking by cancer. Oncogene 27: 6939–6957. 10.1038/onc.2008.345 19029936

[20] Yang J, Liu Y (2001) Dissection of key events in tubular epithelial to myofibroblast transition and its implications in renal interstitial fibrosis. Am J Pathol 159: 1465–1475. 11583974 10.1016/S0002-9440(10)62533-3 PMC1850509

[21] Lemma S, Karihtala P, Haapasaari KM, Jantunen E, Soini Y, et al (2013) Biological roles and prognostic values of the epithelial-mesenchymal transition-mediating transcription factors Twist, ZEB1 and Slug in diffuse large B-cell lymphoma. Histopathology 62: 326–333. 10.1111/his.12000 23190132

[22] Sanchez-Tillo E, Fanlo L, Siles L, Montes-Moreno S, Moros A, et al (2014) The EMT activator ZEB1 promotes tumor growth and determines differential response to chemotherapy in mantle cell lymphoma. Cell Death Differ 21: 247–257. 10.1038/cdd.2013.123 24013721 PMC3890947

[23] Goldstein B, Macara IG (2007) The PAR proteins: fundamental players in animal cell polarization. Dev Cell 13: 609–622. 17981131 10.1016/j.devcel.2007.10.007 PMC2964935

[24] Royer C, Lu X (2011) Epithelial cell polarity: a major gatekeeper against cancer? Cell Death Differ 18: 1470–1477. 10.1038/cdd.2011.60 21617693 PMC3178423

[25] Zeisberg M, Neilson EG (2009) Biomarkers for epithelial-mesenchymal transitions. J Clin Invest 119: 1429–1437. 10.1172/JCI36183 19487819 PMC2689132

[26] Baum B, Georgiou M (2011) Dynamics of adherens junctions in epithelial establishment, maintenance, and remodeling. J Cell Biol 192: 907–917. 10.1083/jcb.201009141 21422226 PMC3063136

[27] Mikami S, Katsube K, Oya M, Ishida M, Kosaka T, et al (2011) Expression of Snail and Slug in renal cell carcinoma: E-cadherin repressor Snail is associated with cancer invasion and prognosis. Lab Invest 91: 1443–1458. 10.1038/labinvest.2011.111 21808237

[28] Yu Q, Zhang K, Wang X, Liu X, Zhang Z (2010) Expression of transcription factors snail, slug, and twist in human bladder carcinoma. J Exp Clin Cancer Res 29: 119 10.1186/1756-9966-29-119 20809941 PMC2942802

[29] Peinado H, Ballestar E, Esteller M, Cano A (2004) Snail mediates E-cadherin repression by the recruitment of the Sin3A/histone deacetylase 1 (HDAC1)/HDAC2 complex. Mol Cell Biol 24: 306–319. 14673164 10.1128/MCB.24.1.306-319.2004 PMC303344

[30] Shin NR, Jeong EH, Choi CI, Moon HJ, Kwon CH, et al (2012) Overexpression of Snail is associated with lymph node metastasis and poor prognosis in patients with gastric cancer. BMC Cancer 12: 521 10.1186/1471-2407-12-521 23151184 PMC3552976

[31] Gottwein E, Cullen BR (2010) A human herpesvirus microRNA inhibits p21 expression and attenuates p21-mediated cell cycle arrest. Journal of virology 84: 5229–5237. 10.1128/JVI.00202-10 20219912 PMC2863803

[32] Takahashi E, Funato N, Higashihori N, Hata Y, Gridley T, et al (2004) Snail regulates p21(WAF/CIP1) expression in cooperation with E2A and Twist. Biochemical and biophysical research communications 325: 1136–1144. 15555546 10.1016/j.bbrc.2004.10.148

[33] Cavallin LE, Goldschmidt-Clermont P, Mesri EA (2014) Molecular and cellular mechanisms of KSHV oncogenesis of Kaposi's sarcoma associated with HIV/AIDS. PLoS pathogens 10: e1004154 10.1371/journal.ppat.1004154 25010730 PMC4092131

[34] de Oliveira DE, Ballon G, Cesarman E (2010) NF-kappaB signaling modulation by EBV and KSHV. Trends in microbiology 18: 248–257. 10.1016/j.tim.2010.04.001 20452220

[35] Werb Z (1997) ECM and cell surface proteolysis: regulating cellular ecology. Cell 91: 439–442. 9390552 10.1016/s0092-8674(00)80429-8

[36] Kessenbrock K, Plaks V, Werb Z (2010) Matrix metalloproteinases: regulators of the tumor microenvironment. Cell 141: 52–67. 10.1016/j.cell.2010.03.015 20371345 PMC2862057

[37] Jia YL, Shi L, Zhou JN, Fu CJ, Chen L, et al (2011) Epimorphin promotes human hepatocellular carcinoma invasion and metastasis through activation of focal adhesion kinase/extracellular signal-regulated kinase/matrix metalloproteinase-9 axis. Hepatology 54: 1808–1818. 10.1002/hep.24562 22045676

[38] Takeshita H, Yoshizaki T, Miller WE, Sato H, Furukawa M, et al (1999) Matrix metalloproteinase 9 expression is induced by Epstein-Barr virus latent membrane protein 1 C-terminal activation regions 1 and 2. J Virol 73: 5548–5555. 10364303 10.1128/jvi.73.7.5548-5555.1999 PMC112612

[39] Jha HC, Upadhyay SK, M AJP, Lu J, Cai Q, et al (2013) H2AX phosphorylation is important for LANA-mediated Kaposi's sarcoma-associated herpesvirus episome persistence. J Virol 87: 5255–5269. 10.1128/JVI.03575-12 23449797 PMC3624323

[40] Sun Z, Jha HC, Robertson ES (2015) Bub1 in Complex with LANA Recruits PCNA To Regulate Kaposi's Sarcoma-Associated Herpesvirus Latent Replication and DNA Translesion Synthesis. Journal of virology 89: 10206–10218. 10.1128/JVI.01524-15 26223641 PMC4580184

[41] Uppal T, Jha HC, Verma SC, Robertson ES (2015) Chromatinization of the KSHV Genome During the KSHV Life Cycle. Cancers 7: 112–142. 10.3390/cancers7010112 25594667 PMC4381254

[42] Jha HC, Lu J, Verma SC, Banerjee S, Mehta D, et al (2014) Kaposi's sarcoma-associated herpesvirus genome programming during the early stages of primary infection of peripheral blood mononuclear cells. mBio 5. 10.1128/mBio.02261-14 PMC4271552 25516617

[43] Sun Z, Xiao B, Jha HC, Lu J, Banerjee S, et al (2014) Kaposi's sarcoma-associated herpesvirus-encoded LANA can induce chromosomal instability through targeted degradation of the mitotic checkpoint kinase Bub1. Journal of virology 88: 7367–7378. 10.1128/JVI.00554-14 24741095 PMC4054434

[44] Lu J, Jha HC, Verma SC, Sun Z, Banerjee S, et al (2014) Kaposi's sarcoma-associated herpesvirus-encoded LANA contributes to viral latent replication by activating phosphorylation of survivin. Journal of virology 88: 4204–4217. 10.1128/JVI.03855-13 24478433 PMC3993767

[45] Lu J, Verma SC, Cai Q, Robertson ES (2011) The single RBP-Jkappa site within the LANA promoter is crucial for establishing Kaposi's sarcoma-associated herpesvirus latency during primary infection. J Virol 85: 6148–6161. 10.1128/JVI.02608-10 21507979 PMC3126528

[46] Ebnet K, Suzuki A, Horikoshi Y, Hirose T, Meyer Zu Brickwedde MK, et al (2001) The cell polarity protein ASIP/PAR-3 directly associates with junctional adhesion molecule (JAM). EMBO J 20: 3738–3748. 11447115 10.1093/emboj/20.14.3738 PMC125258

[47] Hung TJ, Kemphues KJ (1999) PAR-6 is a conserved PDZ domain-containing protein that colocalizes with PAR-3 in Caenorhabditis elegans embryos. Development 126: 127–135. 9834192 10.1242/dev.126.1.127

[48] Cai Q, Xiao B, Si H, Cervini A, Gao J, et al (2012) Kaposi's sarcoma herpesvirus upregulates Aurora A expression to promote p53 phosphorylation and ubiquitylation. PLoS Pathog 8: e1002566 10.1371/journal.ppat.1002566 22396649 PMC3291660

[49] Jha HC, Lu J, Saha A, Cai Q, Banerjee S, et al (2013) EBNA3C-mediated regulation of aurora kinase B contributes to Epstein-Barr virus-induced B-cell proliferation through modulation of the activities of the retinoblastoma protein and apoptotic caspases. Journal of virology 87: 12121–12138. 10.1128/JVI.02379-13 23986604 PMC3807909

[50] Jha HC, Yang K, El-Naccache DW, Sun Z, Robertson ES (2015) EBNA3C regulates p53 through induction of Aurora kinase B. Oncotarget 6: 5788–5803. 25691063 10.18632/oncotarget.3310 PMC4467402

[51] Nelson WJ (2009) Remodeling epithelial cell organization: transitions between front-rear and apical-basal polarity. Cold Spring Harb Perspect Biol 1: a000513 10.1101/cshperspect.a000513 20066074 PMC2742086

[52] Kataoka H, Murayama T, Yokode M, Mori S, Sano H, et al (2000) A novel snail-related transcription factor Smuc regulates basic helix-loop-helix transcription factor activities via specific E-box motifs. Nucleic acids research 28: 626–633. 10606664 10.1093/nar/28.2.626 PMC102498

[53] Escriva M, Peiro S, Herranz N, Villagrasa P, Dave N, et al (2008) Repression of PTEN phosphatase by Snail1 transcriptional factor during gamma radiation-induced apoptosis. Molecular and cellular biology 28: 1528–1540. 10.1128/MCB.02061-07 18172008 PMC2258777

[54] Kaufhold S, Bonavida B (2014) Central role of Snail1 in the regulation of EMT and resistance in cancer: a target for therapeutic intervention. Journal of experimental & clinical cancer research: CR 33: 62. 25084828 10.1186/s13046-014-0062-0 PMC4237825

[55] Lin F, Wang N, Zhang TC (2012) The role of endothelial-mesenchymal transition in development and pathological process. IUBMB life 64: 717–723. 10.1002/iub.1059 22730243

[56] Keller SA, Hernandez-Hopkins D, Vider J, Ponomarev V, Hyjek E, et al (2006) NF-kappaB is essential for the progression of KSHV- and EBV-infected lymphomas in vivo. Blood 107: 3295–3302. 16380446 10.1182/blood-2005-07-2730 PMC1432097

[57] Hastak K, Gupta S, Ahmad N, Agarwal MK, Agarwal ML, et al (2003) Role of p53 and NF-kappaB in epigallocatechin-3-gallate-induced apoptosis of LNCaP cells. Oncogene 22: 4851–4859. 12894226 10.1038/sj.onc.1206708

[58] Dzeng RK, Jha HC, Lu J, Saha A, Banerjee S, et al (2015) Small molecule growth inhibitors of human oncogenic gammaherpesvirus infected B-cells. Molecular oncology 9: 365–376. 10.1016/j.molonc.2014.09.006 25306391 PMC4305020

[59] Wu Y, Deng J, Rychahou PG, Qiu S, Evers BM, et al (2009) Stabilization of snail by NF-kappaB is required for inflammation-induced cell migration and invasion. Cancer cell 15: 416–428. 10.1016/j.ccr.2009.03.016 19411070 PMC2881229

[60] Rodriguez-Pelaez M, Fernandez-Garcia MS, Gutierrez-Corral N, de Francisco R, Riestra S, et al (2010) Kaposi's sarcoma: an opportunistic infection by human herpesvirus-8 in ulcerative colitis. J Crohns Colitis 4: 586–590. 10.1016/j.crohns.2010.03.006 21122564

[61] Ye F, Lei X, Gao SJ (2011) Mechanisms of Kaposi's Sarcoma-Associated Herpesvirus Latency and Reactivation. Adv Virol 2011. 10.1155/2011/193860 PMC3103228 21625290

[62] Cain RJ, Ridley AJ (2009) Phosphoinositide 3-kinases in cell migration. Biol Cell 101: 13–29. 10.1042/BC20080079 19055486

[63] Li Q, Zhou F, Ye F, Gao SJ (2008) Genetic disruption of KSHV major latent nuclear antigen LANA enhances viral lytic transcriptional program. Virology 379: 234–244. 10.1016/j.virol.2008.06.043 18684478 PMC2626151

[64] Cai QL, Knight JS, Verma SC, Zald P, Robertson ES (2006) EC5S ubiquitin complex is recruited by KSHV latent antigen LANA for degradation of the VHL and p53 tumor suppressors. PLoS Pathog 2: e116 17069461 10.1371/journal.ppat.0020116 PMC1626105

[65] Liu J, Martin HJ, Liao G, Hayward SD (2007) The Kaposi's sarcoma-associated herpesvirus LANA protein stabilizes and activates c-Myc. J Virol 81: 10451–10459. 17634226 10.1128/JVI.00804-07 PMC2045471

[66] Shamay M, Liu J, Li R, Liao G, Shen L, et al (2012) A protein array screen for Kaposi's sarcoma-associated herpesvirus LANA interactors links LANA to TIP60, PP2A activity, and telomere shortening. J Virol 86: 5179–5191. 10.1128/JVI.00169-12 22379092 PMC3347335

[67] Si H, Robertson ES (2006) Kaposi's sarcoma-associated herpesvirus-encoded latency-associated nuclear antigen induces chromosomal instability through inhibition of p53 function. J Virol 80: 697–709. 16378973 10.1128/JVI.80.2.697-709.2006 PMC1346846

[68] Xiao B, Verma SC, Cai Q, Kaul R, Lu J, et al (2010) Bub1 and CENP-F can contribute to Kaposi's sarcoma-associated herpesvirus genome persistence by targeting LANA to kinetochores. J Virol 84: 9718–9732. 10.1128/JVI.00713-10 20660191 PMC2937805

[69] Li SY, Davidson PJ, Lin NY, Patterson RJ, Wang JL, et al (2006) Transport of galectin-3 between the nucleus and cytoplasm. II. Identification of the signal for nuclear export. Glycobiology 16: 612–622. 16473834 10.1093/glycob/cwj089

[70] Sahni SK (2007) Endothelial cell infection and hemostasis. Thromb Res 119: 531–549. 16875715 10.1016/j.thromres.2006.06.006

[71] Xu Y, Ganem D (2007) Induction of chemokine production by latent Kaposi's sarcoma-associated herpesvirus infection of endothelial cells. J Gen Virol 88: 46–50. 17170435 10.1099/vir.0.82375-0

[72] Zheng G, Lyons JG, Tan TK, Wang Y, Hsu TT, et al (2009) Disruption of E-cadherin by matrix metalloproteinase directly mediates epithelial-mesenchymal transition downstream of transforming growth factor-beta1 in renal tubular epithelial cells. Am J Pathol 175: 580–591. 10.2353/ajpath.2009.080983 19590041 PMC2716958

[73] Micalizzi DS, Farabaugh SM, Ford HL (2010) Epithelial-mesenchymal transition in cancer: parallels between normal development and tumor progression. J Mammary Gland Biol Neoplasia 15: 117–134. 10.1007/s10911-010-9178-9 20490631 PMC2886089

[74] Batlle E, Sancho E, Franci C, Dominguez D, Monfar M, et al (2000) The transcription factor snail is a repressor of E-cadherin gene expression in epithelial tumour cells. Nat Cell Biol 2: 84–89. 10655587 10.1038/35000034

[75] Jin H, Yu Y, Zhang T, Zhou X, Zhou J, et al (2010) Snail is critical for tumor growth and metastasis of ovarian carcinoma. Int J Cancer 126: 2102–2111. 10.1002/ijc.24901 19795442

[76] Kosaka T, Kikuchi E, Mikami S, Miyajima A, Shirotake S, et al (2010) Expression of snail in upper urinary tract urothelial carcinoma: prognostic significance and implications for tumor invasion. Clin Cancer Res 16: 5814–5823. 10.1158/1078-0432.CCR-10-0230 20947514

[77] Storrs CH, Silverstein SJ (2007) PATJ, a tight junction-associated PDZ protein, is a novel degradation target of high-risk human papillomavirus E6 and the alternatively spliced isoform 18 E6. J Virol 81: 4080–4090. 17287269 10.1128/JVI.02545-06 PMC1866151

[78] McCaffrey LM, Montalbano J, Mihai C, Macara IG (2012) Loss of the Par3 polarity protein promotes breast tumorigenesis and metastasis. Cancer Cell 22: 601–614. 10.1016/j.ccr.2012.10.003 23153534 PMC3500525

[79] Cai Q, Verma SC, Lu J, Robertson ES (2010) Molecular biology of Kaposi's sarcoma-associated herpesvirus and related oncogenesis. Adv Virus Res 78: 87–142. 10.1016/B978-0-12-385032-4.00003-3 21040832 PMC3142360

[80] Saha A, Halder S, Upadhyay SK, Lu J, Kumar P, et al (2011) Epstein-Barr virus nuclear antigen 3C facilitates G1-S transition by stabilizing and enhancing the function of cyclin D1. PLoS Pathog 7: e1001275 10.1371/journal.ppat.1001275 21347341 PMC3037348

[81] Grotegut S, von Schweinitz D, Christofori G, Lehembre F (2006) Hepatocyte growth factor induces cell scattering through MAPK/Egr-1-mediated upregulation of Snail. EMBO J 25: 3534–3545. 16858414 10.1038/sj.emboj.7601213 PMC1538570

[82] Lu J, Verma SC, Murakami M, Cai Q, Kumar P, et al (2009) Latency-associated nuclear antigen of Kaposi's sarcoma-associated herpesvirus (KSHV) upregulates survivin expression in KSHV-Associated B-lymphoma cells and contributes to their proliferation. J Virol 83: 7129–7141. 10.1128/JVI.00397-09 19439469 PMC2704763

[83] Lee SH, Lee SJ, Jung YS, Xu Y, Kang HS, et al (2009) Blocking of p53-Snail binding, promoted by oncogenic K-Ras, recovers p53 expression and function. Neoplasia 11: 22–31, 26p following 31. 19107228 10.1593/neo.81006 PMC2606115

