# Expression of Concern: KSHV-Mediated Regulation of Par3 and SNAIL Contributes to B-Cell Proliferation

**DOI:** 10.1371/journal.ppat.1010480

**Published:** 2022-04-11

**Authors:** The PLOS Pathogens Editors

Following the publication of this article [1], concerns were raised regarding the results presented in Figs 1, 3, 5, 7, and 8. Specifically,

- The Par3 and GAPDH and panel of Fig 1C appear similar to the Par3 panel and GAPDH panel of Fig 7F.
- The BJAB LANA panel of Fig 3A appears to be devoid of any signal or background noise.
- In Fig 3D, the DAPI panels for PBMC-UN and PBMC-INF do not appear to demonstrate the DAPI signal present in the corresponding Merge panels.
- In Fig 5A, the following panels appear more similar than would be expected from independent samples:
  ○ The first panel of Day 1 and the first panel of Day 2.
  ○ The second panel of Day 1 and the second panel of Day 2.
  ○ The third panel of Day 1 and the fourth panel of Day 2.
  ○ The fourth panel of Day 1 and the third panel of Day 2.
- In the SNAIL panel of Fig 7G there appear to be horizonal and vertical irregularities surrounding the band representing the BC3-sh-c results.
- The tumor sizes reported in Fig 8B appear to exceed sizes commonly accepted for mouse tumor studies. The authors did not provide scientific or ethical justification for the tumor sizes presented in the article.

**Fig 5:**
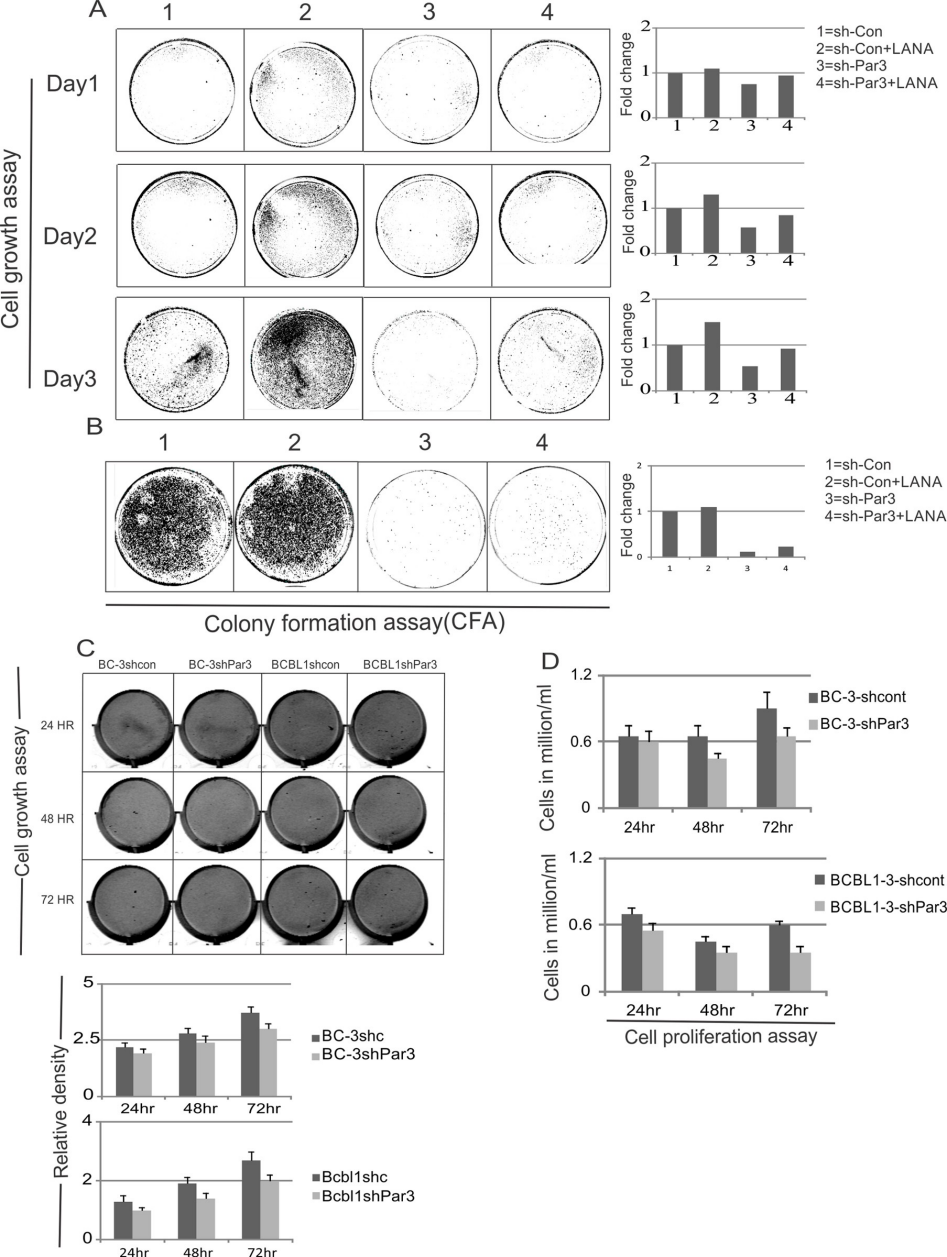
Par3 knockdown leads to delay in cell proliferation. (A) Cell growth assay was carried out in HEK-293 cells. As indicated plasmids were transiently transfected in corresponding plates. 48 hour post transfection, 50,000 cells were plated for all groups into 100 mm dishes. Graphs are presented on the basis of intensity of colonies. Li-Cor Odyssey Scanner was used for scanning these plates. (B) Colony formation assays were carried out under puromycin and G418 antibiotic selection using HEK-293 cell lines. Further cell density was scanned after fixing with 3% PFA and staining with crystal violet. Representative graphs were also plotted for every set of experiments. Quantitation was done on the basis of intensity of colonies in every plates. (C) Cell growth assays were carried out in BC-3 and BCBL1 cells. As indicated plasmids were transiently transfected and transferred to corresponding flasks. 48 hour post transfection, 50,000 cells were plated for all groups into 6 well plates. Further cell densities were determined. Graphs are presented on the basis of intensity of cells. Li-Cor Odyssey Scanner was used for scanning the plates. Plotted graph based on cell density. (D) Cell proliferation assays were performed by using Trypan blue staining. 48 hour transfection, cells were counted for day 1, 2 and 3 and plotted for live cells accordingly.

The corresponding author states that the Par3 and the GAPDH panels presented in Figs 1C and 7F appear similar because these panels represent the same experimental conditions, and clarified that the Merged and DAPI panels for PBMC-UN and PBMC-INF in Fig 3D have been swapped inadvertently during figure preparation. Furthermore, the corresponding author disagrees with the journal’s assessment of the concerns with the BJAB LANA panel of Fig 3A and the SNAIL panel of Fig 7G. The underlying data for the Fig 7G SNAIL panel is provided in the S5 Fig below. The authors have not provided an updated Fig 3. The journal has not received underlying data files for the results presented in Figs 1, 3 and 7 other than those data presented in the S5 Fig.

Regarding Fig 5, the corresponding author explains that the results for Days 1, 2, and 3 were taken from the same plates, and clarified that the PFA fixing and crystal violet staining described in the figure legend for Fig 5A applied to the plates presented in Fig 5B only. The figure legend for Fig 5 has been updated to correct this error. Furthermore, the corresponding author clarified that the panels 3 and 4 for the Day 2 results have been swapped inadvertently during figure preparation. The corresponding author’s explanation that the results presented in Fig 5A were captured from the same plates on subsequent days satisfactorily explains why the results presented in Days 1 and 2 appear similar for respective panels, but it raises further concerns that the patterns of cell density presented in the Day 3 panels do not appear to match the patterns presented in their respective Day 1 and Day 2 results, as would be expected from results obtained from the same plates on subsequent dates. The updated Fig 5 below has been provided by the authors, but the journal did not receive underlying data files to support the results presented in Fig 5.

The corresponding author disagrees with the concerns raised regarding the tumor sizes reported in Fig 8B. They state that the animals used in this study were monitored daily and indicated that they would immediately proceed with euthanising if the weight of the mouse with tumor growth and ascites exceeded 20% of the initial weight, or if any of the animals were in distress at any point. However, the authors did not provide individual level data on tumor measurements requested by the journal, but instead the corresponding author stated that sometimes it is possible that a 40 to 50% increase in tumor size may occur within a 24-hour interval. In the absence of individual level data reporting the tumor measurements the journal remains concerned that the tumors reported Fig 8B exceed sizes commonly accepted for mouse tumor studies. The authors have not provided underlying data files to support the results presented in Fig 8.

The *PLOS Pathogens* Editors issue this expression of concern to notify readers of the above issues.

## Supporting information

S5 Fig Original data underlying the Fig 7G SNAIL results. (TIF)
