# Supplementary figures and images for: KSHV-Mediated Regulation of Par3 and SNAIL Contributes to B-Cell Proliferation
Source: PLoS Pathog. 2016 Jul 27;12(7):e1005801. doi: 10.1371/journal.ppat.1005801 (PMC4963126; doi:10.1371/journal.ppat.1005801)

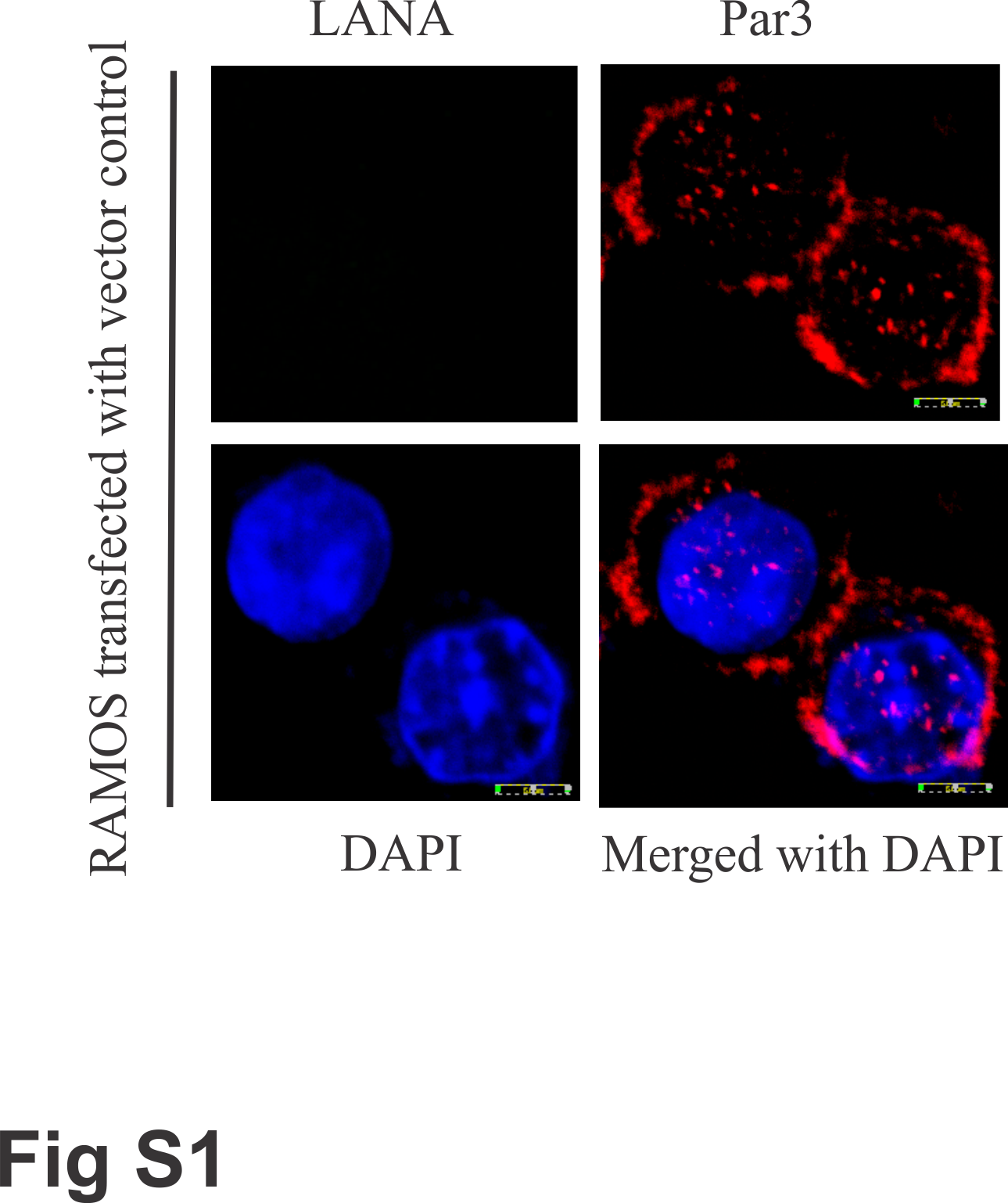

Supplement: S1 Fig — Ramos cells were transiently transfected with vector alone and Par3 in the absence of LANA. Par3 staining was predominantly localized at the cell periphery and inner cytoplasmic membrane. (TIF) [file ppat.1005801.s001.tif]

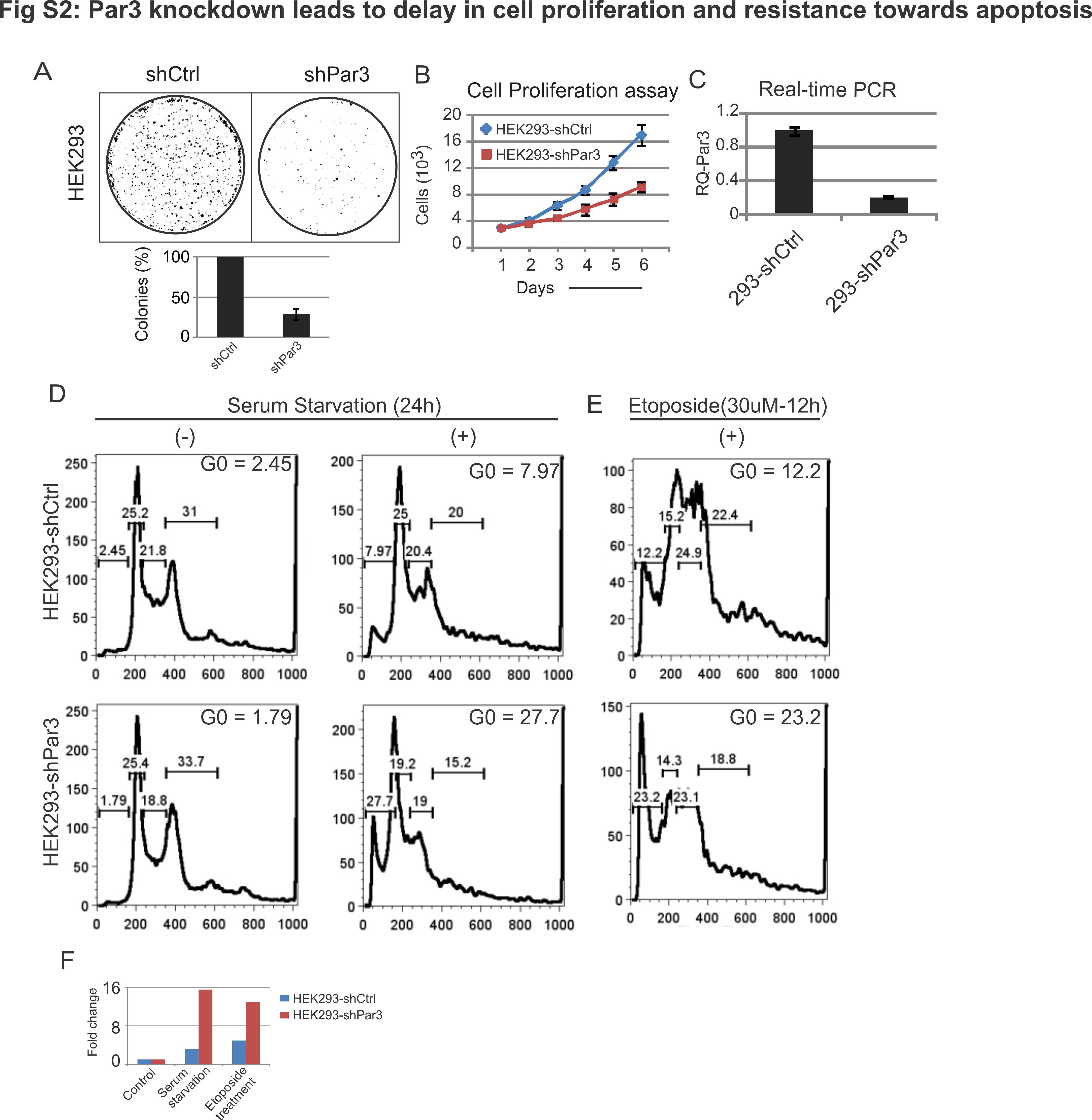

Supplement: S2 Fig — (A) Colony formation assays were carried out under puromycin antibiotic selection using HEK-293 cell lines. Representative graphs were also plotted for every set of experiments. Quantification was based on the % of colonies as 100 in control plates compared to the Par3 knockdown. (B) Cell proliferation assays monitored using cell counting from day 1 to 6 in HEK-293 cells. Graphs were drawn as number of cells per thousand for sh-control and sh-Par3 -BAC-KSHV cells. (C) Real-time PCR was performed to determine the efficiency of Par3 knockdown at the transcript levels. (D) HEK-293 cells were used to study the resistance from apoptosis after serum starvation. sh-Par3 and sh-control plasmids were transfected for 24 hr followed by serum starvation for 24 hr. Propidium iodide (PI) staining was performed with flow cytometry. Graphs represents the phases of the cell cycle. G0 phase of cells were determined as cell death population. (E) HEK-293 cells were transfected with sh-control and sh-Par3 for 24 hr followed by 12 hr etoposide treatment. PI staining was examined by flow cytometry. (F) A representative graph in fold change explaining the cell population in G0 phase either in serum starvation or etoposide treatment compared to control for HEK293-shControl and HEK293-shPar3. (TIF) [file ppat.1005801.s002.tif]

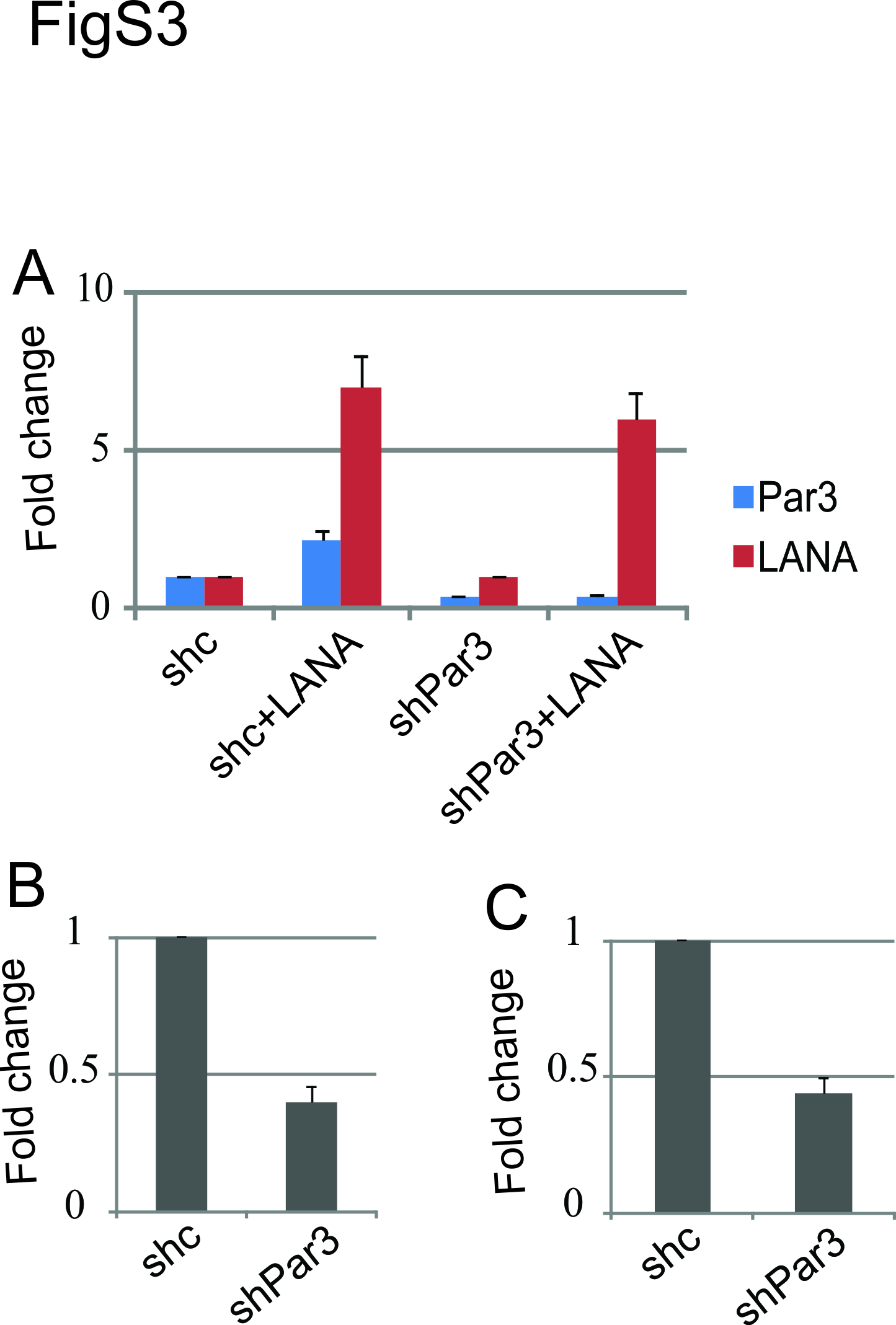

Supplement: S3 Fig — (A) LANA and Par3 expression were checked for LANA and Par3 in exogenous expressed transfected cells for LANA and Par3 sh construct. GAPDH was used as endogenous control. (B and C). Par3 expression was assessed in BC-3 and BCBL1 cells transfected with Par3sh and control. GAPDH was used as endogenous control. (TIF) [file ppat.1005801.s003.tif]

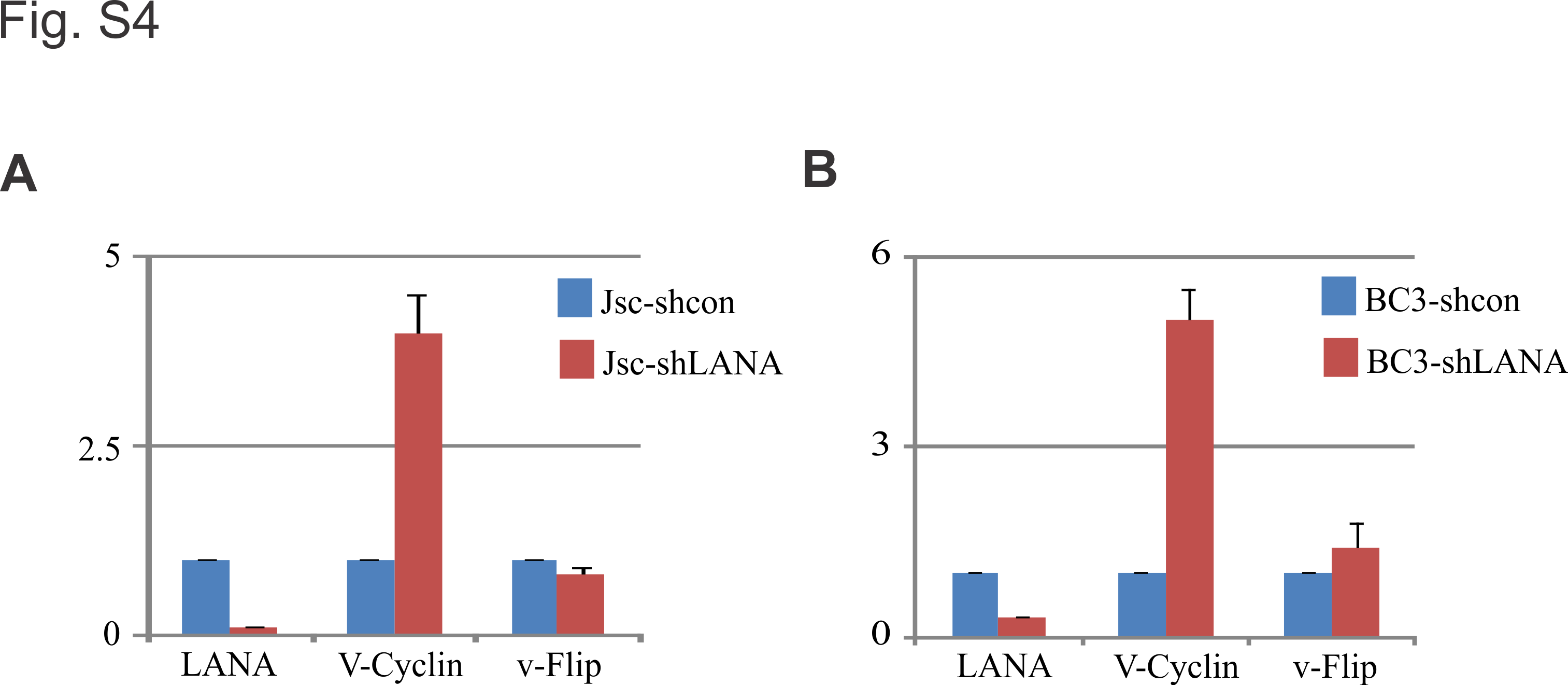

Supplement: S4 Fig — (A-B) BC-3 and JSC-1 LANA knockdown compared to vector control cells were evaluated for LANA, v-Cyclin and v-Flip transcript expression. qRT-PCR was performed with cDNA samples. (TIF) [file ppat.1005801.s004.tif]
