# Supplementary figures and images for: Expression of Concern: KSHV-Mediated Regulation of Par3 and SNAIL Contributes to B-Cell Proliferation
Source: PLoS Pathog. 2022 Apr 11;18(4):e1010480. doi: 10.1371/journal.ppat.1010480 (PMC9000101; doi:10.1371/journal.ppat.1010480)

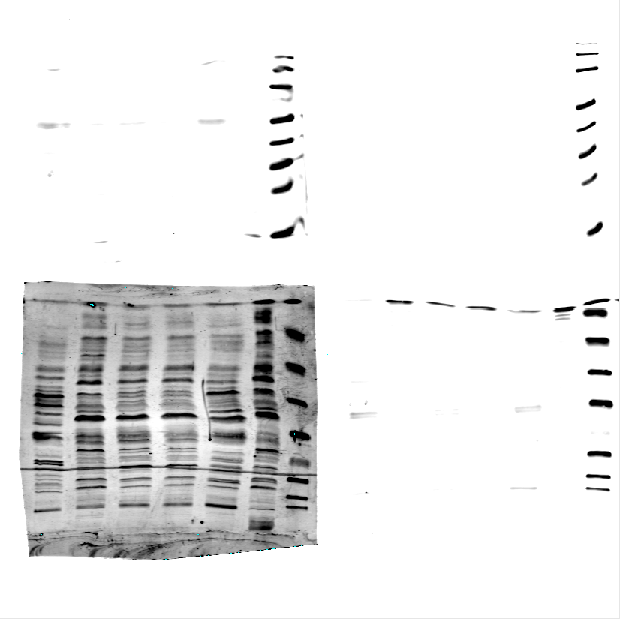

Supplement: S5 Fig — (TIF) [file ppat.1010480.s001.tif]
